# Symmetry and the Role of the Anion Sublattice in Aurivillius Oxyfluoride Bi_2_TiO_4_F_2_

**DOI:** 10.1021/acs.inorgchem.1c01933

**Published:** 2021-09-01

**Authors:** Andrew T. Giddings, Euan A. S. Scott, Martin C. Stennett, David C. Apperley, Colin Greaves, Neil C. Hyatt, Emma E. McCabe

**Affiliations:** † Department of Materials Science and Engineering, The University of Sheffield, Mappin Street, Sheffield, S1 3JD, U.K.; ‡ School of Physical Sciences, University of Kent, Canterbury, Kent CT2 7NH, U.K.; § Department of Chemistry, Durham University, South Road, Durham DH1 3LE, U.K.; ∥ School of Chemistry, The University of Birmingham, Edgbaston, Birmingham B15 2TT, U.K.; ⊥ Department of Physics, Durham University, South Road, Durham DH1 3LE, U.K.

## Abstract

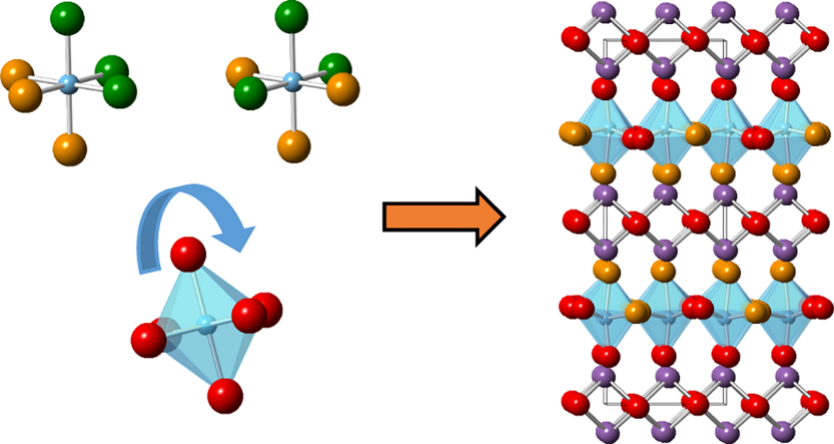

The photocatalytic and dielectric behaviors of Aurivillius oxyfluorides such as Bi_2_TiO_4_F_2_ depend sensitively on their crystal structure and symmetry but these are not fully understood. Our experimental work combined with symmetry analysis demonstrates the factors that influence anion order and how this might be tuned to break inversion symmetry. We explore an experimental approach to explore anion order, which combines Rietveld analysis with strain analysis.

## Abstract 2

Aurivillius oxyfluoride Bi_2_TiO_4_F_2_ shows a promising photocatalytic behavior, which has been related to its polar structure, but polar properties have been difficult to reproduce in bulk samples. We explore a possible anion order and how this could give polar structures when combined with other distortions. We highlight the influence of strain on anion order and therefore the properties in these systems.

## 1 Introduction

The renewed interest in mixed anion materials, containing two or more anions, results from both their increased compositional degrees of freedom as well as the opportunity to use anion order to tune the electronic structure, symmetry, and related properties.^1−5^ These “designer materials”^6^ include photocatalysts,^7,8^ magnetic,^9,10^ superconducting,^11^ and nonlinear optical materials,^12,13^ and ferroelectrics.^14^ These properties are acutely sensitive to structural features, including the anion sublattice, and so it is crucial to understand anion ordering and the factors that influence it as well as how to characterize it when it arises.

Oxyfluorides, containing both oxide O^2–^ and fluoride F^–^ ions, illustrate these challenges. Topotactic fluorination reactions of layered oxides^15,16^ typically result in oxyfluorides with F^–^ occupation of (otherwise vacant) interstitial sites, which is often accompanied by occupation of one or two anion sites in an ordered fashion. These reactions have been powerful for tuning properties including magnetism, superconductivity, and properties associated with the lack of inversion symmetry.^10,11,17^ However, the similar sizes of O^2–^ and F^–^ ions^18^ mean that single-step solid-state reactions (forming the oxyfluoride directly from oxide and fluoride reagents, often at high temperatures) can result in a lack of long-range order of O^2–^ and F^–^ ions over the anion sites.^19^ The similar X-ray and neutron scattering lengths of O^2–^ and F^–^ ions^20^ make exploring this anion order (whether it is long-range or short-range) particularly challenging.^21^ Theory work highlighting the opportunity to control the symmetry (and therefore properties) using the anion order^4^ has motivated further work on these systems.

**Figure 1:**
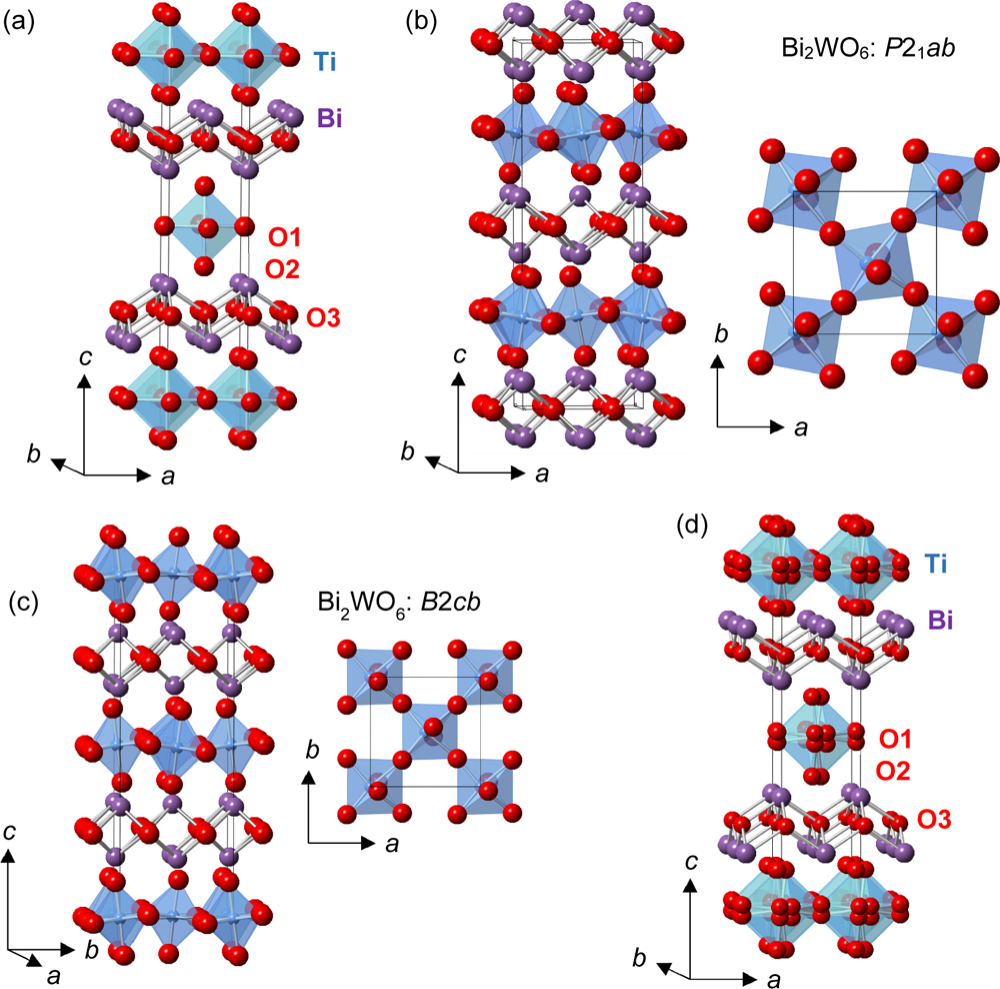
Illustration of (a) ideal ordered *I*4/*mmm* structure for an *n* = 1 Aurivillius phase; (b) low-temperature *P*2_1_*ab* phase for Bi_2_WO_6_ and (c) intermediate *B*2*cb* phase for Bi_2_WO_6_;^30^ (d) disordered *I*4/*mmm* structure of Bi_2_TiO_4_F_2_ (with displacive disorder of equatorial and apical anion positions to 16*n* and 16*m* sites, respectively) from 100 K NPD Rietveld refinement; TiX_6_ or WO_6_ polyhedra, Bi, and O are shown in blue, purple, and red, respectively.

Aurivillius materials, a class of layered perovskite-related materials, have long been known for their ferroelectric properties.^22,23^ They have a general formula Bi_2_*A*_*n*–1_*B*_*n*_*X*_3*n*+3_ (*X* is an oxide or halide ion) and adopt structures based on fluorite-like [Bi_2_O_2_]^2+^ layers separated by blocks of corner-linked *BX*_6_ octahedra, which are *n* layers thick, Figure 1. The *n* = 1 phases Bi_2_WO_6_ and Bi_2_MoO_6_ are perhaps illustrative of many features of this family, adopting polar structures (of *P*2_1_*ab* symmetry) at low temperatures, with rotations of *B*O_6_ octahedra about both the long (out-of-plane) axis and about an in-plane axis, with polar displacements of *B* cations toward the edge of the octahedra.^24−29^ On warming, they undergo a phase transition to a second polar phase of *B*2*cb* symmetry (in which rotations about the long axis are frozen out) before more complex phase transitions occur above their ferroelectric *T*_C_.^30−32^ In addition to their ferroelectric behavior, both are effective photocatalysts, particularly in nanostructured forms.^33−36^

The *n* = 1 Aurivillius oxyfluorides Bi_2_NbO_5_F and Bi_2_TaO_5_F,^37^ Bi_2_VO_5_F,^38^ Bi_2_TiO_4_F_2_,^39^ and Bi_2_CoO_2_F_4_^40^ are less well understood but are increasingly attracting attention for their photocatalytic behavior,^41−44^ and this is thought to be influenced by their polar and dielectric properties.^45^ However, the dielectric behavior of Bi_2_TiO_4_F_2_ is still not fully understood: it was first considered as a ferroelectric^46,47^ with a ferroelectric Curie temperature *T*_C_ = 284 K,^47^ but later, experimental work on bulk ceramic samples found no evidence of ferroelectricity or of a polar crystal structure.^39^ Interestingly, studies on thin films of Bi_2_TiO_4_F_2_ suggest a *T*_C_ of 240 K.^48^ These ambiguities in the literature suggest some sample dependence of properties (and therefore structure and symmetry) that may be sensitive to synthesis routes, cooling rates, and strain. This sensitivity may result from the complexity of the anion sublattice—both in terms of the distribution of O^2–^ and F^–^ ions over the anion sites and any displacive disorder of the anion positions. It is timely to reconsider these materials and, in particular, the anion ordering possibilities and their consequences for symmetry and polar properties.^49^ Our experimental work (based on property measurements as well as neutron powder diffraction (NPD) and electron diffraction) is consistent with earlier studies,^39^ and our symmetry analysis demonstrates that it is possible to break inversion symmetry in these systems by ordering of O^2–^ and F^–^ ions over the anion sites and in combination with octahedral rotations. We explore an experimental method that may be applied more widely to investigate anion order in oxyfluorides based on the Rietveld method combined with global instability index (GII) calculations to introduce structural strain information into structural refinements.

## 2 Methods

Synthesis of Bi_2_TiO_4_F_2_ was achieved by solid-state reaction of a stoichiometric ratio of BiF_3_, Bi_2_O_3_, and TiO_2_. An intimate mixture of reagents was pressed into several 8 mm diameter pellets, which were wrapped in Pt foil and sealed in a quartz tube, together with a separate 0.2 mol excess of BiF_3_, also wrapped in Pt foil (to compensate for BiF_3_ volatilization). Samples were reacted three times at 640 °C for 48 h, with a 2 °C min^–1^ ramp rate; pellets were recovered after each reaction and ground to a fine powder, prior to forming new green pellets for the subsequent reaction. By using this method, it was found possible to produce sintered bodies of ∼82% theoretical density. Herein, we report results from three sample preparations:

- Sample A: determined to comprise 95.5(1)% Bi_2_TiO_4_F_2_ and 4.5(1)% BiOF by weight, according to Rietveld analysis of X-ray and neutron diffraction data (see below). This material was used for acquisition of X-ray, neutron, and electron diffraction data and electrical property measurements.
- Sample B was single-phase Bi_2_TiO_4_F_2_ within the limit of sensitivity of powder X-ray diffraction data.
- Sample C: determined to comprise 88.8(5) % Bi_2_TiO_4_F_2_ and 11.2(5)% BiOF by weight but with evidence of a further trace of an unidentified impurity phase(s) according to Rietveld analysis of powder X-ray diffraction data.

Electrical measurements were performed on sintered pellets (Sample A), with sputtered gold electrodes, using an HP 4192A impedance analyzer with a He cryocooler (Oxford instruments Model CC1.5). Data were corrected for sample geometry prior to analysis. Elemental analysis was performed using a CAMECA SX51 WDS-EPMA; the sintered specimens were embedded in a cold setting epoxy resin and polished to a finish of 0.25 μm diamond paste prior to analysis. Fluorine determination was performed using an F-selective electrode after fusion with Na_2_CO_3_/K_2_CO_3_ and acid digestion.

Variable temperature time-of-flight NPD data were acquired using the high-resolution powder diffractometer HRPD at the ISIS Neutron and Muon Source (Chilton, UK). A 7 g sample was contained in a vanadium can, mounted within a cryostat (Sample A). For the purpose of structure refinement, data collection times were ∼4 h at 300, 260, and 175 K and ∼8 h at 100 K. The diffraction data were normalized to the incident beam spectrum and corrected for detector efficiency (using a vanadium standard) and sample attenuation. Rietveld refinements were carried out using the GSAS suite of programs^50,51^ and TopasAcademic^52,53^ using NPD data from both the high-resolution back scattering detectors (Bank 1: 2θ ≈ 168°, Δ*d*/*d* = 5 × 10^–4^) and moderate-resolution transverse detectors (Bank 2: 2θ ≈ 90°, Δ*d*/*d* = 2 × 10^–3^).

MAS NMR experiments were performed at 300 K, using a Varian Unity Inova spectrometer operating at 282.09 MHz for ^19^F (Samples B and C). The experimental parameters were acquisition time 25 ms, recycle delay 5 s, and spectral width 200 kHz. Chemical shifts were referenced with respect to the signal from CFCl_3_ at *d* = 0.00 ppm.

## 3 Results

### 3.1 Synthesis and Preliminary Analysis

Rietveld refinement using room temperature XRPD data of Sample A was consistent with the formation of an *n* = 1 Aurivillius phase and was fitted with a main phase of *I*4/*mmm* symmetry for Bi_2_TiO_4_F_2_ (95.5(1)% by weight) and a trace of BiOF (4.5(1)% by weight) (see Supporting Information). Elemental analysis by EPMA gave a composition of Bi_1.98(2)_Ti_1.04(2)_O_4_F_2.1(1)_ for Sample A, in good agreement with the target stoichiometry, which is assumed in the subsequent analysis. Electron diffraction data from Sample A were collected at ∼300 K and at ∼100 K, and again, indexed reflections were consistent with the aristotype *I*4/*mmm* model (see Supporting Information). Diffraction patterns taken down the [100] zone axis were indexed using this high-symmetry unit cell, and no additional reflections (or diffuse scattering) were observed. However, further zone axes (e.g. [110] and [001]) would be needed to rule out the possibility of orthorhombic distortions as observed, for example, for Bi_2_NbO_5_F.^54^

### 3.2 Property Measurements

**Figure 2:**
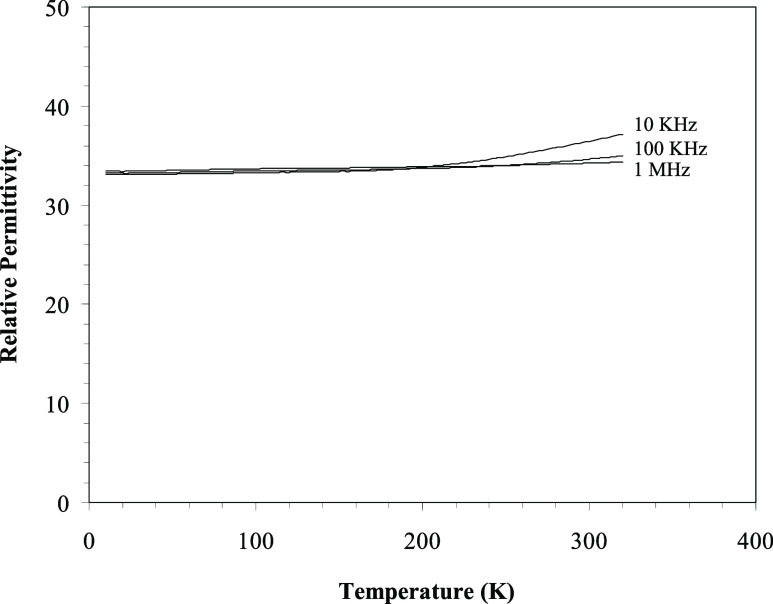
Temperature and frequency dependence of relative permittivity of Bi_2_TiO_4_F_2_ measured between 10 kHz and 1 MHz.

The sintered pellets of Bi_2_TiO_4_F_2_ were highly insulating at room temperature and below. The relative permittivity of Bi_2_TiO_4_F_2_ was measured at 10 kHz to 1 MHz, on pellets of ∼85% theoretical density, prepared from Sample A (95.5(1)% Bi_2_TiO_4_F_2_ and 4.5(1)% BiOF by weight). The relative permittivity was found to be essentially temperature and frequency independent between 10 and 320 K, and there is no evidence for a dielectric anomaly in this temperature range (Figure 2). This contrasts with the report of a paraelectric–ferroelectric phase transition in Bi_2_TiO_4_F_2_ at *T*_C_ = 284 K by Ismailzade and Ravez.^47^

### 3.3 ^19^F Magic Angle Spinning Solid-State NMR

^19^F magic angle spinning solid-state nuclear magnetic resonance (^19^F MAS ssNMR) data were collected for two samples of Bi_2_TiO_4_F_2_:

- Sample B was single phase within the limit of sensitivity of XRPD.
- Sample C contained 11.2(5)% BiOF by weight and a further unidentified impurity phase as determined from Rietveld analysis of XRPD data.

**Figure 3:**
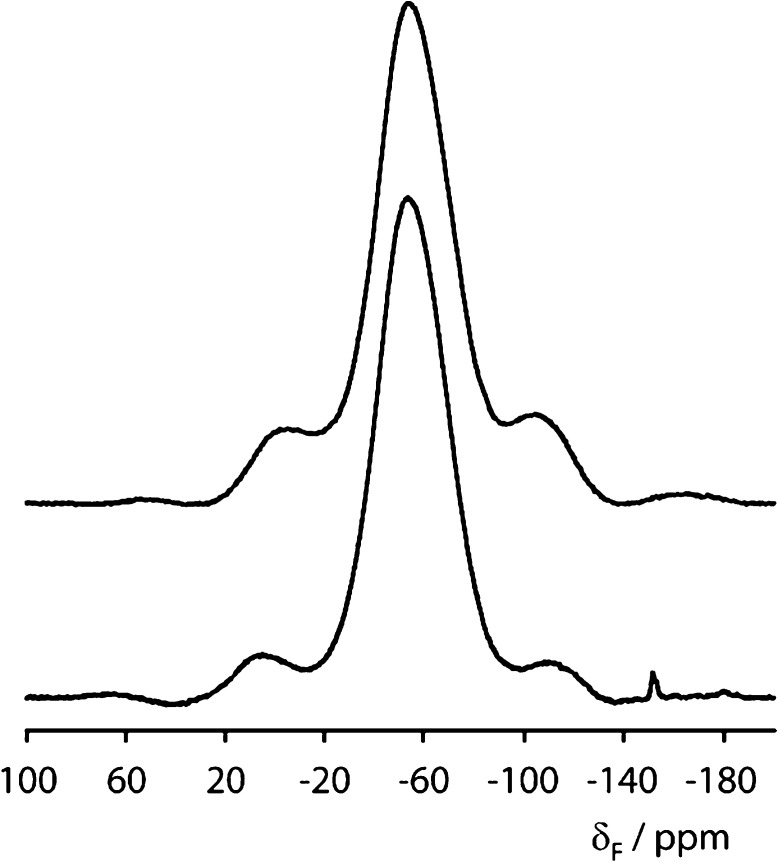
^19^F MAS NMR spectra of two specimens of Bi_2_TiO_4_F_2_; Sample B, lower, was determined to be single phase, within the limit of sensitivity of powder X-ray diffraction. Sample C, upper, contained an impurity of 11.2(5) wt % BiOF plus an additional unidentified phase. The spin rate was 16.3 KHz, with 200 free induction decays for Sample A and 248 for Sample B.

The spectra from both samples were essentially identical, with a single broad band centered at approximately −54 ppm and associated spinning side bands centered at approximately 0 and −105 ppm, as shown in Figure 3. These spectra are very similar in appearance to the ^19^F NMR spectrum of Bi_2_TiO_4_F_2_ reported by Needs et al.^39^

The broad line width of the ^19^F MAS NMR signals could arise from one or more causes, including

(i) Homonuclear (^19^F, ^19^F) dipolar coupling between F atoms in close proximity.
(ii) Heteronuclear coupling, for example, between ^19^F and quadrupolar ^209^Bi (100% natural abundance).
(iii) Static and/or dynamic disorder of ^19^F atoms over the available anion sites.

Heteronuclear dipolar coupling effects were considered too small to give rise to the observed broad line widths previously observed in Bi_2_TiO_4_F_2_ and Bi_2_NbO_5_F,^39^ although we cannot rule out some coupling between bismuth and F in the apical *X*2 site. This suggests that disorder of the anion sublattice, principally displacive disorder of the anion sites, (and disorder of F^–^/O^2–^ over the available sites to a lesser extent) is a cause of the broad line shape. Needs et al. presented ^19^F MAS NMR for both Bi_2_NbO_5_F (with most likely only one F^–^ per Nb*X*_6_ polyhedron) and Bi_2_TiO_4_F_2_ (with two F^–^ per Ti*X*_6_ polyhedron), and it is striking that the spectrum for Bi_2_NbO_5_F is at least as broad as that observed for Bi_2_TiO_4_F_2_.^39^ This could indicate that the displacive disorder of the anion sites (e.g. displacement of the equatorial and apical sites off their high symmetry 4*c* and 4*e* sites in the *I*4/*mmm* model to 16*n* and 16*m*, respectively, as discussed below) may be sufficient to explain the broadened signal. We cannot rule out F^–^ occupancy of more than one site (e.g. partial occupancy of equatorial and apical sites) which could also give rise to some broadening of the signal. It is noted that the signal from Sample B is broader than that from Sample C (when measured at the same spin rate). This suggests that the fluorine atoms are more strongly coupled, that is, on average, closer together, in Sample B compared to Sample C. This could suggest that antisite disorder (i.e. F^–^ occupancy of equatorial and apical sites), which is likely to depend on the preparation method and thermal history, could also play a role in broadening the NMR signal. (In the case of Sample C, changing the spin rate from 14.0 to 16.3 kHz resulted in some narrowing of the signal, suggesting that homonuclear coupling is at least partly responsible for the broad line shape.) There are relatively few ^19^F NMR studies on titanium oxyfluorides, making it difficult to compare the chemical shift observed here with typical shifts for terminal and bridging fluoride sites in Ti(O,F)_6_ octahedra. The presence of a BiOF impurity in Sample C (noted from analysis of powder diffraction data) means that ∼13% of the fluoride content is in this BiOF impurity phase, in which it occupies a bonding position very similar to the apical *X*2 site in Bi_2_TiO_4_F_2_. This particular BiOF environment does not give rise to an apparently distinctive signal in our ^19^F MAS NMR data above presumably because it coincides with the main resonance.

### 3.4 Structural Analysis Using Neutron Powder Diffraction Data

#### 3.4.1 Rietveld Refinements and the Average Structure

NPD data were collected at 100 K and at 300 K (i.e. below and above the reported *T*_C_ of 284 K^47^) to investigate possible structural changes within this temperature range; this study utilized Sample A which was also characterized by X-ray and electron diffraction (Section 3.1) and electrical measurements (Section 3.2). No attempt was made to distinguish between oxygen and fluorine sites due to their similar neutron scattering lengths (5.803 and 5.654 fm for oxygen and fluorine, respectively),^20^ and all anion sites were modeled as occupied by oxygen. Data collected at all temperatures were qualitatively similar with the main peaks consistent with the aristotype model of *I*4/*mmm* symmetry for an *n* = 1 Aurivillius phase. Several additional very weak reflections were also observed, some of which were fitted by traces of BiOF (∼4%), consistent with lab XRPD analysis. Attempts to include other impurity phases (such as TiO_2_, Bi_2_O_3_, BiF_3_, Bi_3_Ti_2_O_8_F, and Bi_7_F_11_O_5_) to fit other peaks were unsuccessful.

**Figure 4:**
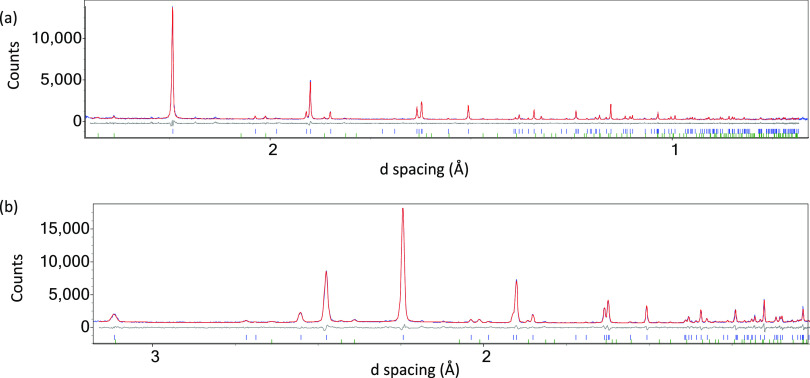
Rietveld refinement profiles using 100 K NPD data and the disordered model of *I*4/*mmm* symmetry for Bi_2_TiO_4_F_2_ (with displacive disorder of equatorial and apical anion positions to 16*n* and 16*m* sites, respectively) with the upper (blue) ticks showing peak positions for the main phase (96.1(3)% by weight) and the lower (green) ticks showing peak positions for the BiOF impurity (3.9(3)% by weight); *R*_wp_ = 6.39% and *R*_p_ = 5.85% (55 parameters). The upper panel (a) shows data from the backscattered band and the lower panel (b) shows data from the 90° bank. Observed, calculated, and difference profiles are shown in blue, red, and grey, respectively.

Rietveld refinement at 100 K using a model of *I*4/*mmm* symmetry gives unfeasibly large atomic displacement parameters (ADPs) for the equatorial anion site *X*1 (and, to a lesser extent, the apical anion site *X*2), (5.6(1) × 100 Å^2^ and 3.7(1) × 100 Å^2^, respectively; see Supporting Information). Allowing these ADPs to refine anisotropically revealed significant displacements (either static or dynamic) of *X*1 along [100] (consistent with rotation of Ti*X*_6_ octahedra about [001]) and along [001] (consistent with rotation about an in-plane axis), similar to the displacive disorder observed in related systems.^55,56^ Moving the equatorial and apical anions to lower symmetry sites (*X*1 from 4*c* to 16*n* and *X*2 from 4*e* to 16*m*) gave more reasonable ADPs (1.2(1) × 100 Å^2^ and 0.82(9) × 100 Å^2^, respectively) and an improvement in fit [*R*_wp_ decreased from 6.95% (52 parameters) for the ordered model to 5.67% (55 parameters) for the displacive disordered model]. Refinement details and selected bond lengths are given in Table 1 and refinement profiles in Figure 4, and the disordered structural model of *I*4/*mmm* symmetry is illustrated in Figure 1d. ADPs were not refined anisotropically for this displacively disordered model.

**Table 1:** Details from Rietveld Refinements Using NPD Data and the Disordered Model of *I*4/*mmm* Symmetry for Bi_2_TiO_4_F_2_ (with Displacive Disorder of Equatorial and Apical Anion Positions to 16*n* and 16*m* Sites, Respectively) and Selected Bond Lengths and Angles

		100 K	175 K	260 K	300 K
*R*_wp_ (%)		5.67	4.73	4.66	4.34
*R*_p_ (%)		5.56	4.32	4.16	3.97
*a* (Å)		3.80050(6)	3.80313(4)	3.80705(4)	3.80922(4)
*c* (Å)		16.2990(3)	16.3105(2)	16.3247(2)	16.3328(2)
volume (Å^3^)		235.419(9)	235.913(6)	236.604(6)	236.991(6)
Bi (4*e*)	*z*	0.32815(7)	0.32830(6)	0.32825(7)	0.32820(6)
	*U*_iso_ × 100 (Å^2^)	1.4(1)	1.61(7)	1.83(7)	2.08(7)
Ti (2*a*)	*U*_iso_ × 100 (Å^2^)	2.4(1)	2.4(1)	2.7(1)	3.0(1)
*X*1 (16*n*)	*x*	0.0706(9)	0.0699(7)	0.0685(8)	0.0665(8)
	*z*	0.0154(2)	0.0160(2)	0.0160(2)	0.0163(2)
	*U*_iso_ × 100 (Å^2^)	1.2(1)	1.3(1)	1.5(1)	1.7(1)
*X*2 (16*m*)	*x*	0.051(1)	0.0505(9)	0.0491(9)	0.0515(8)
	*z*	0.1166(1)	0.1166(1)	0.1166(1)	0.1165(1)
	*U*_iso_ × 100 (Å^2^)	1.6(1)	2.2(1)	2.4(1)	2.4(1)
*X*3 (4*d*)	*U*_iso_ × 100 (Å^2^)	0.82(9)	1.06(6)	1.20(7)	1.39(7)
Bi–*X*2 (Å)		1 × 2.574(5)	1 × 2.579(5)	1 × 2.589(5)	1 × 2.580(4)
Bi–*X*2 (Å)		2 × 2.848(1)	2 × 2.8486(9)	2 × 2.8510(9)	2 × 2.8545(9)
Bi–*X*2 (Å)		1 × 3.097(5)	1 × 3.095(5)	1 × 3.090(5)	1 × 3.105(5)
Bi–*X*3 (Å)		4 × 2.2876(7)	4 × 2.2906(6)	4 × 2.2924(6)	4 × 2.2932(6)
Ti–*X*1 (Å)		4 × 1.9355(9)	4 × 1.9377(7)	4 × 1.9389(8)	4 × 1.9396(7)
Ti–*X*2 (Å)		2 × 1.920(5)	2 × 1.921(2)	2 × 1.921(2)	2 × 1.923(2)

Similar models were used for Rietveld refinements using 175 K, 260 K, and 300 K NPD data and showed little change within this temperature range. The *X*1 and *X*2 sites again had unusually large ADPs for the ordered *I*4/*mmm* model (*U*_iso_ values for *X*1 and *X*2 sites of 5.8(1) × 100 Å^2^ and 4.0(1) × 100 Å^2^ at 175 K, 6.0(1) × 100 Å^2^ and 4.1(1) × 100 Å^2^ at 260 K, and 5.9(1) × 100 Å^2^ and 4.2(1) × 100 Å^2^ at 300 K, respectively). There is relatively little temperature dependence in these values, which may indicate a static (displacive) disorder rather than a dynamic disorder. The disordered models of *I*4/*mmm* symmetry gave more reasonable ADPs and are most appropriate to describe the long-range structure within this temperature range (Table 1).

The high ADPs may indicate local/short-range distortions reflected by our disordered *I*4/*mmm* model (i.e. with displacive disorder of the equatorial and apical anion sites) or could indicate that rotations of the Ti*X*_6_ octahedra are coherent over longer length scales and give an average structure of lower symmetry. It can be helpful to consider possible lower symmetry structures in terms of the high-symmetry parent structure with symmetry-lowering distortions (described by an irreducible representation or irrep) imposed on it.^57,58^ Common distortions in *n* = 1 Ruddlesden-Popper and Aurivillius phases include rotations of the *BX*_6_ octahedra. These rotations can be about the long axis of the unit cell described by the X_2_^+^ irrep (or *a*^0^*a*^0^*c*^±^ rotations in Glazer notation or 00θ 00θ in Aleksandrov’s notation) or about an in-plane axis such as the X_3_^+^ rotations (*a*^–^*a*^–^*c*^0^ in Glazer notation or ΦΦ0 ΦΦ0 in Aleksandrov’s notation). (We have used the *c*^±^ notation to indicate rotation of the *BX*_6_ octahedra about the *c* axis, but we cannot define the direction of rotation with respect to other perovskite-like layers in the block because these are single-layer materials.) These have been explored and tabulated^59,60^ and are explained in more detail in the Supporting Information. Aurivillius materials are well known for their polar and ferroelectric behavior, and this often results from in-plane polar displacements (along [100] or [110] of the high symmetry unit cell) of cations relative to anions described by the Γ_5_^–^ irrep.^27−29^ It is also possible to have an out-of-plane polarization (along the long axis of the unit cell) described by the Γ_3_^–^ irrep. (The same irrep language can also be used to describe anion ordering arrangements, as explored in Section 4.2.) ISODISTORT^57,58^ was used to explore likely structural rotations including rotations of the Ti*X*_6_ octahedra^59,60^ and in-plane displacements (see Supporting Information). Larger, orthorhombic unit cells could index one or two additional weak reflections (e.g. √2 *a* × √2 *a* × *c* cells index a peak at ∼2.14 Å as (107); 2*a* × 2*a* × *c* cells index a peak at ∼2.30 Å as (303); see Supporting Information) and so lower symmetry models with larger unit cells were considered. Mode inclusion analysis^61,62^ suggested that the greatest improvement in fit was observed for models allowing in-plane polar displacements (described by the Γ_5_^–^ irrep; see above and Supporting Information), but polar models did not fit the intensity to the superstructure peaks and often still gave high ADPs. Although an exhaustive search was made to find a lower symmetry model to give a significantly better fit than this disordered *I*4/*mmm* model, the search was unsuccessful: models did not give intensity to additional reflections and/or intensity was predicted where none was observed. Care was also taken to consider models of *Pbca* and *Pca*2_1_ symmetries (as reported for Bi_2_NbO_5_F^54^ or Bi_2_WO_6_^24,28,30^), but the fits from these models were no better than that from the disordered *I*4/*mmm* model, and suggested high ADPs for equatorial (and apical) anion sites. No broadening of *hh*0 or *hhl* reflections (indexed with respect to the *I*4/*mmm* model), which might have suggested an orthorhombic distortion, was observed. This suggests that the best description of the long-range average structure of Bi_2_TiO_4_F_2_ in the temperature range 100—300 K from NPD data is the disordered model of *I*4/*mmm* symmetry (i.e. with displacive disorder of the equatorial and apical anion sites).

#### 3.4.2 Madelung Energy Calculations for Bi_2_TiO_4_F_2_

Madelung energy calculations give a measure of structural stability based on electrostatics (i.e. neglecting polarization and lone pair effects). Calculations on this Bi–Ti—O–F system (see Supporting Information) indicate that the formation of Bi_2_TiO_4_F_2_ (for all anion distributions) is enthalpically favorable. These calculations suggest F occupancy of apical *X*2 and fluorite *X*3 anion sites in preference to equatorial *X*1 sites (Madelung energies of 2.56 × 10^4^ kJ mol^–1^ for equatorial *X*1 = F; 2.68 × 10^4^ kJ mol^–1^ for apical *X*2 = F, and 2.64 × 10^4^ kJ mol^–1^ for fluorite *X*3 = F were calculated). This may reflect the purely ionic nature of these calculations which do not take into account the inert pair effect of Bi^3+^ ions, which is likely to influence the bonding around the fluorite *X*3 and apical *X*2 anion sites.

#### 3.4.3 Anion Distribution in Bi_2_TiO_4_F_2_

The similar neutron scattering lengths of O and F make it difficult to determine the distribution of O^2–^ and F^–^ anions over the anion sites. Bond valence sum analysis^64,65^ has been used to investigate anion ordering in oxyfluorides,^19,39,54^ and analysis by Needs et al. suggests that F^–^ ions in Bi_2_TiO_4_F_2_ are most likely to occupy the equatorial (*X*1) anion sites.^39^ Our analysis using bond lengths from our 300 K disordered *I*4/*mmm* model (see Supporting Information) is less definitive: F^–^ occupation of the *X*1 site gives cation valences closest to those expected but occupation of *X*2 is preferred by the anion BVS values; F^–^ occupation of the *X*3 site (within the fluorite-like Bi_2_*X*_2_ layers) is the least favorable. Overall, the difference between F^–^ occupation of *X*1 (equatorial) and *X*2 (apical) sites is small. The stoichiometry of Bi_2_TiO_4_F_2_ is consistent with F^–^ occupying half the anion sites in the perovskite layers, and as explained by Needs et al.,^39^ there are three possibilities for this: the equatorial *X*1 site could be fully occupied by F^–^, the apical *X*2 site could be fully occupied by F^–^, or both *X*1 and *X*2 sites could be half-occupied by F^–^. It is possible that more than one site is occupied by F^–^ ions and the anion distribution may be sensitive to synthesis temperature, cooling rate, and sample history.

## 4 Discussion

### 4.1 Structural Analysis

Analysis of NPD data, discussed above, indicates displacements of equatorial *X*1 and apical *X*2 anion sites consistent with short-range tilting of Ti*X*_6_ octahedra. However, there is no evidence to suggest long-range ordering of this tilting from NPD or electron diffraction data, consistent with analysis by Needs et al.^39^ Tilting of the *BX*_6_ octahedra in Bi_2_*A*_2_*B*_3_O_12_ and related Aurivillius phases is thought to occur to relieve strain in stacking the wider perovskite and more narrow fluorite-like [Bi_2_O_2_]^2+^ layers (with natural *a* parameter *a*_f_ = 3.80 Å).^66^ In *n* = 1 Aurivillius materials with no *A*-site cations, the ideal width of the perovskite layers, *a*_p_, can be approximated by *a*_p_ = 2(*r*_B_ + *r*_X_), with *r*_X_ being the weighted mean anion radius. For Bi_2_TiO_4_F_2_ (*r*_B_ = *r*_Ti_ = 0.605 Å, *r*_O_ = 1.35 Å, and *r*_F_ = 1.285 Å)^18^ with F^–^ in equatorial *X*1 or apical X2 sites, *a*_p_ = 3.78 Å and *a*_p_ = 3.91 Å, respectively. In terms of lattice mismatch, F occupancy of equatorial *X*1 sites might be expected (giving perovskite blocks of a similar width to the [Bi_2_O_2_]^2+^ layers) but tilting of the Ti*X*_6_ octahedra, about in-plane and out-of-plane axes, can also reduce this interfacial mismatch while maintaining satisfactory Ti–*X* bond lengths. The bond valence sum analysis and our understanding of strain suggest that F occupancy of equatorial *X*1 sites is certainly possible in Bi_2_TiO_4_F_2_ and is consistent with the conclusions of Needs et al.^39^ However, this contrasts with several other *n* = 1 Aurivillius and Ruddlesden-Popper oxyfluorides including Bi_2_NbO_5_F,^54^ Sr_2_ScO_3_F,^67^ and Sr_2_MnO_3_F,^68^ which are reported to have F occupancy of apical anion sites despite similar arguments involving stacking strain between [Bi_2_O_2_]^2+^ and Nb(O,F)_2_ layers (*a*_p_ = 3.95 Å and *a*_p_ = 4.08 Å for F^–^ in equatorial *X*1 or apical *X*2 sites, respectively) or between SrO and Mn(O,F)_2_ layers (ideal *a*_SrO_ ≈ 3.65 Å; for Sr_2_ScO_3_F *a*_p_ = 4.12 Å and *a*_p_ = 4.19 Å for F^–^ in equatorial or apical sites, respectively; for Sr_2_MnO_3_F *a*_p_ = 3.86 Å and *a*_p_ = 3.99 Å for F^–^ in equatorial or apical sites, respectively). It is striking that Bi_2_TiO_4_F_2_ has relatively small stacking strain compared to these examples, suggesting the possibility that anion distribution might be tuned by strain engineering, for example.^69^ Wider consideration of the bonding in Bi_2_TiO_4_F_2_, beyond the purely ionic model, may also be relevant—particularly given the importance of pπ dπ *B*–O bonding (often reflected in shorter *B*–O bonds) that can influence the stereochemistry in oxyfluorides.^19^ Similar factors also influence the N^3–^/O^2–^ order in oxynitrides.^70^

Bond valence sum analysis has been used to investigate possible anion ordering in other oxyfluorides,^19^ but the uncertainty of the anion order in Bi_2_TiO_4_F_2_ from BVS analysis prompted us to examine the use of the more powerful “global instability index” (GII). This is a measure of lattice strain—the extent to which the valence sum rule is broken (based on bond lengths).^71,72^ The GII is defined as
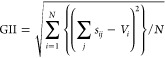
1and it gives a measure of the extent to which the observed bond valence sums at a cation site (*S*_*ij*_) differ from the theoretical cation valence (*V*_*i*_), averaged over all atoms in the formula unit. In general, it is accepted that structures with GII < 0.05 v.u. are essentially unstrained, whereas those with GII > 0.20 v.u. are disposed toward relaxation, or distortion, in order to minimize bond strain. The key point is that the GII can give an indication of the overall structural strain on the O/F distribution. Calculations were first carried out for the ordered *I*4/*mmm* model (i.e. equatorial and apical anions on high-symmetry 4*c* and 4*e* sites) but gave GII values greater than the accepted threshold value of 0.20, regardless of anion arrangement, consistent with at least short-range tilting of Ti*X*_6_ octahedra, as discussed above. The GII was then calculated for the disordered *I*4/*mmm* model of Bi_2_TiO_4_F_2_ (i.e. equatorial and apical anions on lower-symmetry 16*n* and 16*m* sites) for various bonding anion arrangements, assuming full occupancy of all cation sites and using constraints to maintain anion stoichiometry (taking into account the site multiplicities as appropriate)
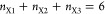
2a
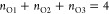
2b
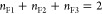
2c

**Figure 5:**
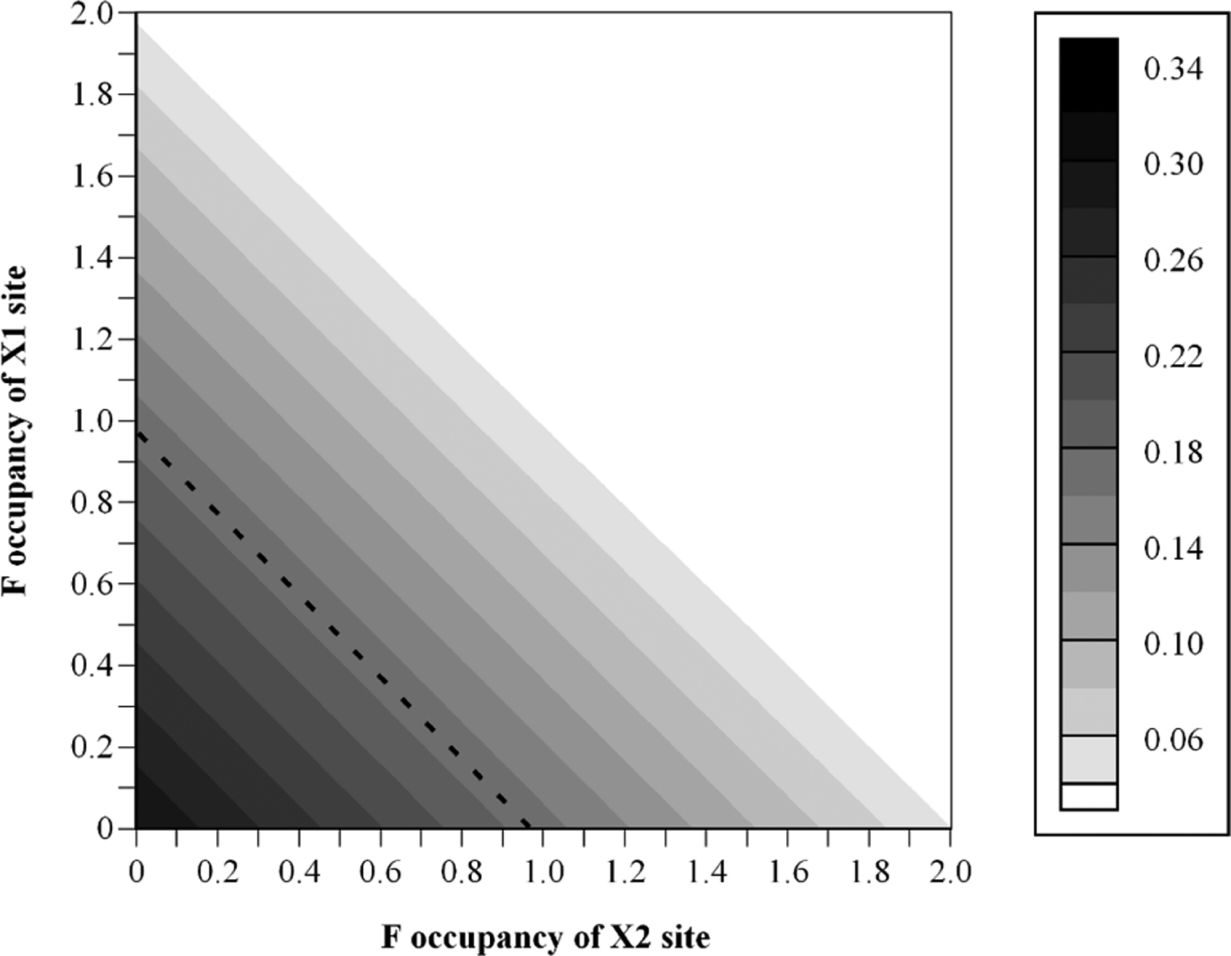
Contour plot showing variation of the GII with F occupation of anion sites in Bi_2_TiO_4_F_2_, assuming that *X*1 and *X*2 anions are displaced to quarter occupied 16*n* (*x*, 0.5, *z*) and 16*m* (*x*, *x*, *z*) general positions, respectively; increasing GII values are correlated with heavier shading. Note: contour plot assumes stoichiometric constraints defined by eqs 2a, 2b, and 2c (the white area lies outside these stoichiometric constraints). The broken line indicates the isoline defining the upper stability limit of the Bi_2_TiO_4_F_2_ structure, with GII = 0.20.

The GII was calculated for all possible O/F distributions at intervals of Δ*n* = 0.1. The results are shown in the form of a contour map in Figure 5. Note that, by virtue of eqs 2a, 2b, and 2c, setting the F occupancy of the *X*1 and *X*2 sites defines the F occupancy of the *X*3 site and the O occupancy of sites *X*1, *X*2, and *X*3.

Figure 5 shows that the GII values for the disordered *I*4/*mmm* models span the range corresponding to essentially unstrained (GII = 0.06) to prohibitively strained (GII > 0.20) structures. Further, the threshold value of GII = 0.20 corresponds to the isoline defined by the inequality given as eq 3 (and shown by the broken line in Figure 5)
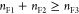
3

This highlights the strain induced if F occupancy of the *X*3 site exceeds that of the *X*1 and *X*2 sites. Importantly, the lowest GII values are calculated for structures with F exclusively occupying *X*1 and *X*2 sites; that is, the lowest GII values correspond to the isoline defined by eq 4
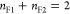
4

However, GII values for the end members of this isoline differ by less than 10% (exclusive F^–^ occupation of the equatorial *X*1 site gives GII = 0.057; exclusive F^–^ occupation of the apical *X*2 site gives GII = 0.062). Using the GII as an indicator of structural strain again suggests that F occupancy of equatorial *X*1 sites is favored, but the difference in strain between *X*1 and *X*2 F occupancy is small, and some partial occupancy would not be unexpected.

### 4.2 Hypothetical Anion-Ordered Models for *n* = 1 Aurivillius and Ruddlesden–Popper Phases

Recent interest in designing polar materials has demonstrated that the anion sublattice can be tuned to break inversion symmetry: (oxidative) topotactic fluorination reactions have been used to tune octahedral tilts to break the inversion symmetry in Ruddlesden–Popper phases,^17^ and theory work has considered anion ordering to give polar heteroanionic units that might crystallize to give polar structures.^4,5^ This prompted us to explore hypothetical O/F ordering patterns in Bi_2_TiO_4_F_2_ more fully, and these models can equally be applied to other *n* = 1 Aurivillius and Ruddlesden-Popper phases which have aristotype structures of *I*4/*mmm* symmetry.

For Bi_2_TiO_4_F_2_ with O^2–^ anions fully occupying *X*3 anion sites in the fluorite layers (and equally for Ruddlesden–Popper phases of *A*_2_*B*O_2_F_2_ stoichiometry), the anion sites in the perovskite layers (equatorial and apical sites) are half-occupied by O and F, giving three possible anion-ordered arrangements (see Supporting Information):

(A) F fully occupies equatorial *X*1 sites (as expected due to stacking strain in Bi_2_TiO_4_F_2_).
(B) F fully occupies apical *X*2 sites.
(C) F and O each half-occupy both equatorial and apical sites.

With the equatorial and apical anion positions being crystallographically distinct sites, ordered arrangements (A) and (B) do not produce any change in the symmetry (it remains centrosymmetric, *I*4/*mmm*). The structure is built up from corner-linked nonpolar TiO_2_F_4/2_ and TiF_2_O_4/2_ octahedra for (A) and (B), respectively (the *x*/*y* notation for the equatorial sites denotes the site of multiplicity *x* shared between *y* cation sites).

**Figure 6:**
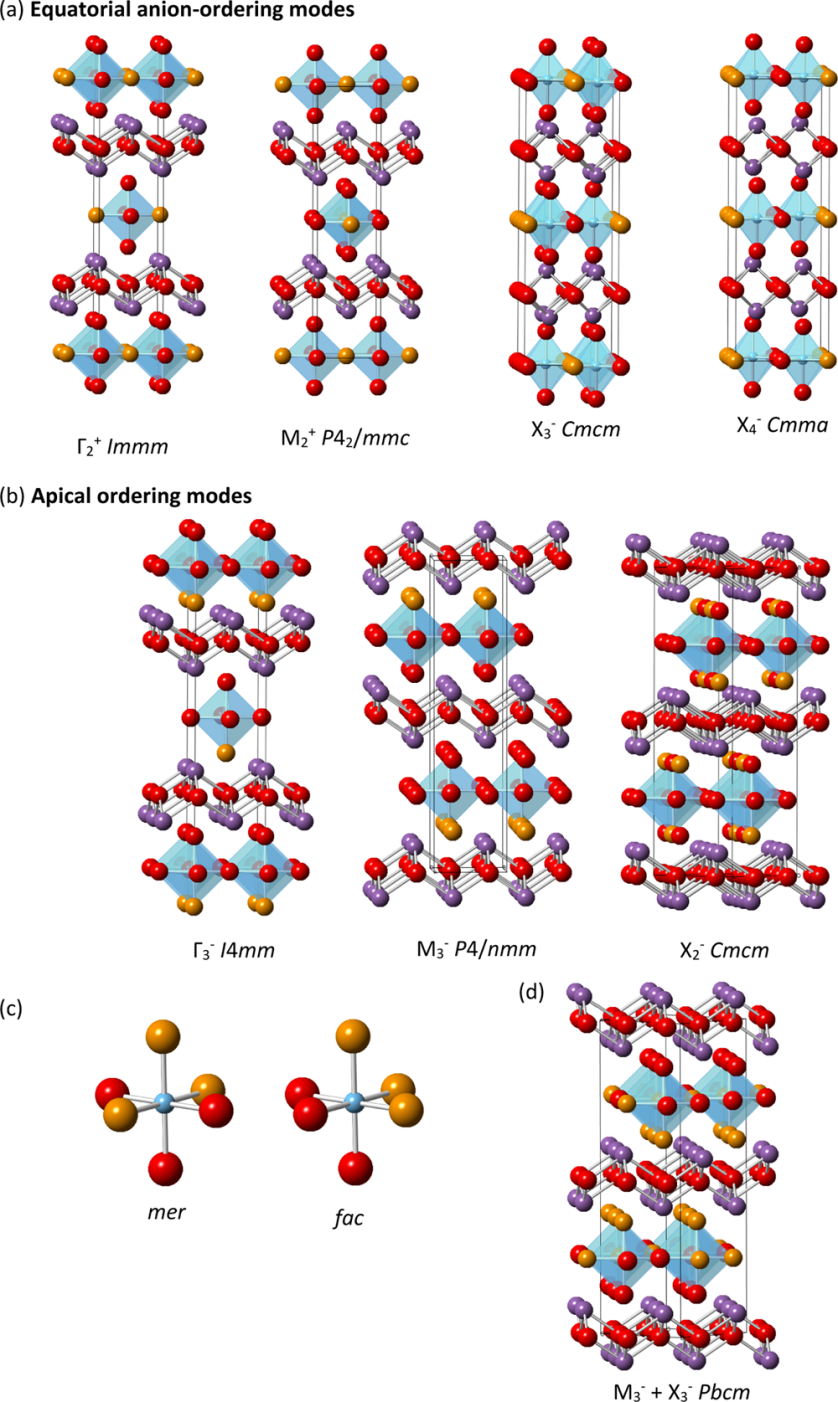
Illustration of (a) equatorial occupancy modes and (b) apical occupancy modes that can be combined to give anion-ordered structures containing *mer*- and *fac*-TiO_3_F_3_ octahedra in Bi_2_TiO_4_F_2_; Bi, Ti, O, and F are shown in purple, blue, red, and orange, respectively. Analysis was carried out using ISODISTORT using the ordered *I*4/*mmm* model as the parent structure; (c) isolated *mer*- and *fac*-TiO_3_F_3_ units and (d) *fac Pbcm* model (derived from M_3_^–^ apical ordering and X_3_^–^ equatorial ordering modes).

The final arrangement, (C), is interesting because it results in a polar Ti(O_1/2_F_1/2_)_ap_(O_2/2_F_2/2_)_eq_ corner-linked octahedra, that is, either *mer-* or *fac-* [TiO_3_F_3_] units (Figure 6c). The bond valence sum and GII analysis described above for Bi_2_TiO_4_F_2_ suggest that while arrangement (A) is most favored, the difference between arrangements (A) and (B) is small and so (C) might occur over short length scales or could be accessed by strain engineering as demonstrated in strontium manganese oxyfluoride films.^69^

The *mer-* or *fac-* [TiO_3_F_3_] heteroanionic units are both polar, and it is interesting to consider the symmetry of different packing arrangements and whether possible anion ordering could break inversion symmetry to give polar structures. As for our exploration of displacive distortions (Section 3.4.1), ISODISTORT^57,58^ was again used to explore occupancy modes that might give rise to anion-ordered arrangements, and these are illustrated in Figure 6:

- Γ_3_^–^, M_3_^–^, and X_2_^–^ modes can give ordered half-occupancy of apical sites by O and F.
- Γ_2_^+^ and M_2_^+^ modes can give ordered half-occupancy of equatorial sites in a trans fashion.
- X_3_^–^ and X_4_^–^ modes can give ordered half-occupancy of equatorial sites in a cis fashion.

Combinations of apical and equatorial anion modes can be combined to give *mer-* and *fac-*TiO_3_F_3_ octahedra with packing of various symmetries (see Table 2 and Supporting Information). Many of these occupancy modes have been explored more generally by Harada et al.,^5^ but we focus here on those possible in *n* = 1 Aurivillius and Ruddlesden–Popper phases.

**Table 2:** Summary of Anion-Ordered Bi_2_TiO_4_F_2_ Structures Containing *mer*- and *fac*-TiO_3_F_3_ Units in Bi_2_TiO_4_F_2_^a^

apical ordering mode	equatorial ordering mode	unit cell symmetry	TiO_3_F_3_ isomerism	optimized GII at 300 K (v. u.)
Γ_3_^–^	Γ_2_^+^	*Imm*2	*mer*	0.24(4)
	M_2_^+^	*P*4_2_*mc*	*mer*	0.29(3)
	X_3_^–^	*Ama*2	*fac*	0.11(2)
	X_4_^–^	*Abm*2	*fac*	0.18(3)
M_3_^–^	Γ_2_^+^	*Pmmn*	*mer*	0.16(1)
	M_2_^+^	*P*4̅*m*2	*mer*	0.15(1)
	X_3_^–^	*Pbcm*	*fac*	0.09(1)
	X_4_^–^	*Pccm*	*fac*	0.15(1)
X_2_^–^	Γ_2_^+^	*C*2/*c*	*mer*	0.15(2)
	M_2_^+^	*Pnna*	*mer*	0.17(2)
	X_3_^–^	*Pnma*	*fac*	0.27(4)
		*P*2_1_/*m*		
	X_4_^–^	*Pbcm*	*fac*	0.24(3)
		*C*2/*m*		

a Analysis was carried out using ISODISTORT using the ordered *I*4/*mmm* model as the parent structure. GII values were calculated from 300 K NPD Rietveld refinement with bond valence penalties to allow refinement of unit cell parameters and atomic coordinates (as allowed by symmetry).

Both *mer*- and *fac*-TiO_3_F_3_ units are polar (the polar axis is along the *C*_4_ axis in *mer*-TiO_3_F_3_ and along the *C*_3_ axis in *fac*-TiO_3_F_3_), and d^0^ Ti^4+^ ions might be expected to displace away from the center of the unit toward the O^2–^ anions.^19^ As highlighted by Withers et al., the challenge in using anion ordering to design polar materials lies in controlling the relative orientations of the polar units^19^ and, in Bi_2_TiO_4_F_2_, only the polar Γ_3_^–^ occupancy mode (which acts on the apical anion sites) gives polar anion-ordered structures in these cases with out-of-plane polarization along the long axis.

Although it is not possible to differentiate between these different O/F ordering patterns using NPD (due to the similar O and F neutron scattering lengths), the symmetry lowering caused by the anion order allows other structural degrees of freedom, such as cation displacements, which NPD may be sensitive to. However, if these are subtle or short-range, they may not always be detected by standard methods (such distortions may give only diffuse scatter or weak superstructure reflections) making it hard to distinguish between numerous similar models.

**Figure 7:**
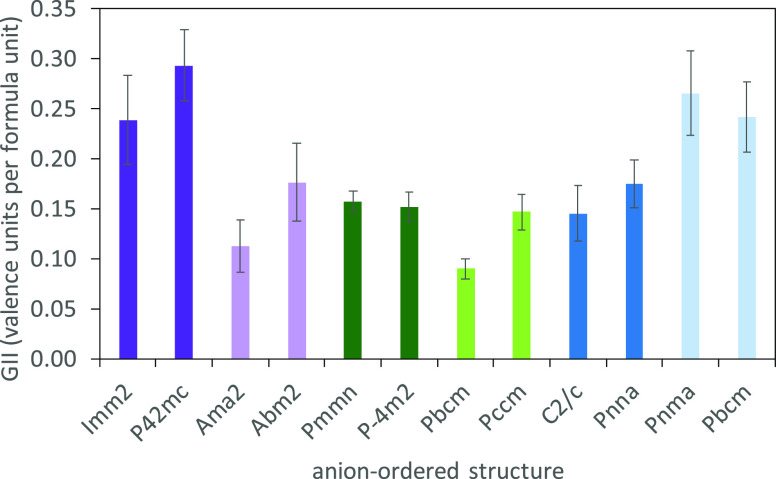
Showing GII values for combined Rietveld and BVS refinements for the anion-ordered models in Table 2; models from Γ_3_^–^ (polar), M_3_^–^, and X_2_^–^ apical ordering modes are shown in purple, green, and blue, respectively, with *mer* and *fac* isomers shown in dark and light shades.

Harada et al. have shown elegantly that the GII can be used to screen potential structures to rule out those with high lattice strain. Their approach involved refining unit cell parameters to minimize the GII (although this could be developed to include lattice degrees of freedom), although it required some constraints on changes in unit cell parameters and the degree of orthorhombic distortion.^5^ Given the recent interest in oxyfluorides and the challenges in investigating anion ordering,^21^ we have built on the GII approach and used the GII as a means of introducing lattice strain information into our Rietveld refinements. This method might be applied more widely to investigate the anion order in heteroanionic materials with similar scattering lengths for the two anions. Structural refinements of the anion-ordered models in Table 2 were carried out using the Rietveld method with additional local subroutines to introduce penalty functions to minimize the difference between the experimental valence and the expected valence for each site (based on bond lengths from the refined structure; see section from the TopasAcademic input file in Supporting Information). This allows the structural model to refine in order to improve the fit to the diffraction data and to minimize bond valence sums, which gives an optimized value for the GII. This is similar to the approach used by Thompson et al.,^74^ but the flexibility of the input files for TopasAcademic^52^ allows the least squares refinements to be carried out within a single program. This approach uses the experimental diffraction data (in this case, 300 K NPD data) to constrain the structural model rather than additional more arbitrary constraints. Nevertheless, by using penalties for both cation and anion sites, most structures refined to give Ti–O bonds shorter than Ti–F bonds. This is consistent with structures reported for NdNiO_2_F,^75^ α-*A*_3_MoO_3_F_3_ (*A* = Rb and K),^1,4^ and other oxyfluorides.^19^ For both Γ_3_^–^ and M_3_^–^ apical ordering, the GII results in Table 2 and Figure 7 suggest that, in general, the *fac* structures are less strained than the equivalent *mer* structures, which is again consistent with the importance of pπ dπ bonding between oxygen and d^0^ cations, favoring *cis* and *fac* isomers with shorter Ti–O bonds.^19^

It is worth noting that *cis-* and *trans-*isomers are similarly favored for oxynitrides (for which N^3–^ is the more covalent ligand).^70^ However, this preference rarely gives long-range N^3–^/O^2–^ order, and instead, a correlated order (comprising *cis-*anion chains) emerges.^76^ This lack of long-range site order is thought to disrupt long-range correlations between any polar displacement in the oxynitrides,^77,78^ limiting the length scale of any polar region and can give polar nanoregions.^79^

### 4.3 Polar Structures from Nonpolar Anion Order and Nonpolar Tilts

The GII values for many of these structures, derived from the ordered *I*4/*mmm* model, are higher than those calculated above for the disordered *I*4/*mmm* model. It is likely that lattice strain would be further relieved in these anion-ordered models by rotation of TiO_3_F_3_ octahedra, even if only over short length scales, and it is interesting to consider the symmetry implications of this (see Supporting Information for full details). For several nonpolar anion-ordered structures in Table 2, allowing a nonpolar octahedral rotation mode (e.g. either about an in-plane axis or about the out-of-plane axis) can break the inversion symmetry and give a polar structure, either with out-of-plane polarization (described by the Γ_3_^–^ irrep), which is somewhat unusual in Aurivillius materials, or with the more commonly observed in-plane polarization (described by the Γ_5_^–^ irrep). This combination of anion order coupled with nonpolar distortions to break the inversion symmetry has been reported for α-*A*_3_MoO_3_F_3_ (*A* = Rb, K)^14^ and provides a further avenue for research into hybrid-improper origins of polar behavior.^80^ The coupling between the rotational modes, polar distortions, and anion ordering is worthy of further study, and we now consider combinations of tilts and anion ordering. The *fac* model of the *Pbcm* symmetry (M_3_^–^ and X_3_^–^ anion ordering modes) has a low GII. Introducing rotations of TiO_3_F_3_ octahedra about the out-of-plane axis (00θ 00θ, X_2_^+^ irrep; see Section 3.4.1 above and Supporting Information) lowers the symmetry to *Pca*2_1_, allowing in-plane polar distortions (Γ_5_–irrep) and rotation of TiO_3_F_3_ octahedra about an in-plane axis (ΦΦ0 ΦΦ0, X_3_^+^ irrep; see Section 3.4.1 above and Supporting Information). This combination of tilts has been reported for other *n* = 1 Aurivillius phases including Bi_2_MoO_6_,^81,82^ Bi_2_WO_6_,^24,28^ and Bi_2_NbO_5_F,^54^ and the in-plane polar displacements along [110]_t_ are frequently observed in Aurivillius materials. Given that this symmetry is compatible with a *fac*-TiO_3_F_3_ structure predicted to have fairly low strain, it would be interesting to explore how such a phase could be realized. Rietveld refinement for Bi_2_TiO_4_F_2_ using this *Pca*2_1_ model with bond valence penalties gives a GII of 0.05(7), comparable to the disordered *I*4/*mmm* model discussed above; although as noted above, the model does not give intensity for the superstructure reflections (see Supporting Information).

The local (rather than long-range) nature of octahedral tilting may result from the lack of long-range order on the anion sublattice, and the extent of any anion order may be very sensitive to precise composition, reaction temperature, and pressure and any annealing and sample cooling rate. This may explain the observed ferroelectric *T*_C_ = 284 K reported by Ismailzade and Ravez,^47^ which was not seen in other studies on polycrystalline samples of Bi_2_TiO_4_F_2_ (here and Needs et al.^39^). Epitaxial methods for thin film growth may provide a means of controlling the anion arrangement, and the choice of substrates may also influence the lattice strain and tilting; these considerations likely explain the ferroelectric transition at ∼240 K reported in thin films of Bi_2_TiO_4_F_2_^48^ and may be relevant to its photocatalytic behavior.^45^

## 5 Conclusions

This work highlights the preferences for F^–^ occupancy in the perovskite anion sites in the photocatalyst Bi_2_TiO_4_F_2_, and our exploration of strain and bonding helps to explain this preference and how it might be tuned. This is particularly relevant to the structure and behavior of this class of materials in thin film and nanostructured forms where strain could well result in different anion distributions to those observed in bulk ceramic samples. Our symmetry analysis demonstrates the possibility of anion order alone breaking inversion symmetry in a small number of cases in Bi_2_TiO_4_F_2_ as well as several possibilities for the anion order combined with nonpolar octahedral rotations to break inversion symmetry. This is similar to the findings by Fry and Woodward^14^ and provides a new avenue to explore in designing hybrid improper polar materials.^80^ The displacive disorder of the anion sites observed in bulk ceramic samples (here and Needs et al.^39^) is likely to disrupt tendencies for O^2–^/F^–^ ordering over the anion sites, but the possibility of local regions that are non-centrosymmetric (similar to recent reports in oxynitrides^79^) has potential for the design of new materials with relaxor-like properties.^83^ Our use of the Rietveld method combined with bond valence penalties^64,65^ can give additional chemical information for the structural analysis of systems with atoms of similar scattering lengths.

## Supporting Information Available

Preliminary XRPD data, electron diffraction, details from Rietveld refinements using further models, symmetry map showing common structural distortions for *A*_2_*BX*_4_ and Bi_2_*BX*_6_ materials, mode inclusion, bond valence sum analysis, results of Madelung energy calculations, symmetry map illustrating space group, excerpt from input file for TopasAcademic software for Rietveld refinement using bond valence penalties, symmetries of anion-ordered structure with tilts of TiO_3_F_3_ octahedra, symmetries of anion-ordered *n* = 1 Aurivillius phases assuming an ideal parent structure , and crystallographic data from refinements using powder diffraction data (PDF)
