# Supplementary material for: Symmetry and the Role of the Anion Sublattice in Aurivillius Oxyfluoride Bi2TiO4F2
Source: Inorg Chem. 2021 Sep 1;60(18):14105–15. doi: 10.1021/acs.inorgchem.1c01933 (PMC8456413; doi:10.1021/acs.inorgchem.1c01933)
Supplement: Supplementary file 1 — ic1c01933_si_001.pdf [file ic1c01933_si_001.pdf]

Symmetry and the role of the anion sublattice in Aurivillius oxyfluoride $Bi_2TiO_4F_2$

Andrew T. Giddings,[1*] Euan A. S. Scott,[2] Martin C. Stennett,[1] David C. Apperley,[3] Colin Greaves,[4] Neil C. Hyatt[1**] and Emma E. McCabe[2,5**]

[1] Department of Materials Science and Engineering, The University of Sheffield, Mappin Street, Sheffield, S1 3JD, UK.

[2] School of Physical Sciences, University of Kent, Canterbury, Kent, CT2 7NH, UK.

[3] Department of Chemistry, Durham University, South Road, Durham, DH1 3LE, UK.

[4] School of Chemistry, The University of Birmingham, Edgbaston, Birmingham, B15 2TT, UK.

[5] Department of Physics, Durham University, South Road, Durham, DH1 3LE, UK.

* joint corresponding authors? n.c.hyatt@sheffield.ac.uk; emma.mccabe@durham.ac.uk

Supporting information:

1) Preliminary analysis using lab XRPD data:

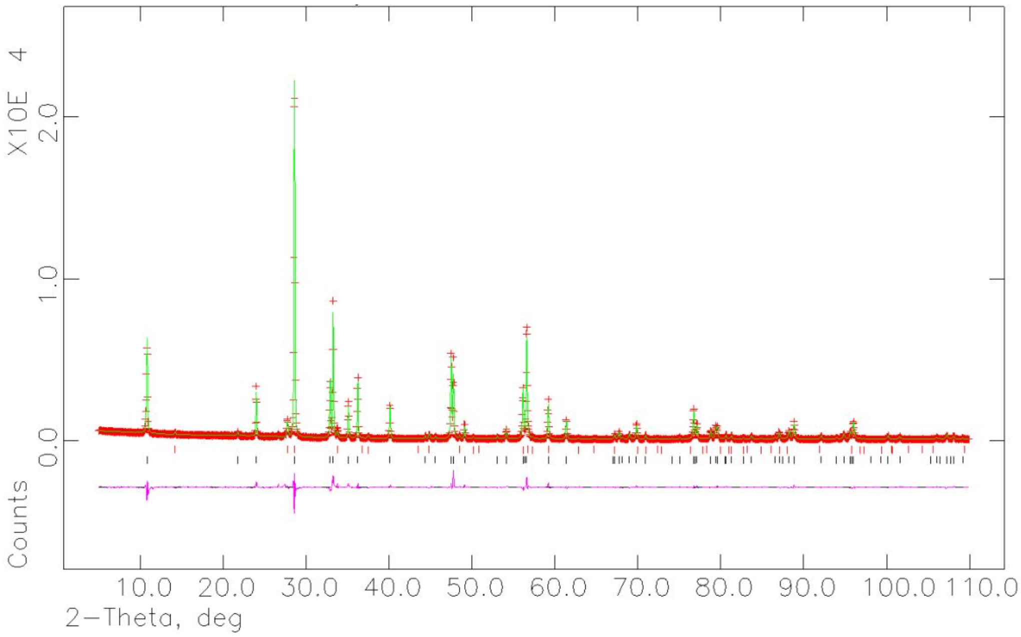

Figure 1: Rietveld refinement profiles using room temperature lab XRPD data for Sample A with a $Bi_2TiO_4F_2$ main phase (of *I*4/*mmm* symmetry, 95.5(1)% by weight, lower black ticks) and a BiOF impurity phase (4.5(1)% by weight); R_{wp} = 9.00%, R_p = 6.89%, χ^2 = 2.86; red crosses, green line and lower pink line show observed, calculated and difference profiles, respectively. XRPD data were collected on a Stoë STADI P diffractometer operating in transmission mode with a monochromated Cu Ka1 radiation source and a position sensitive detector with a step size of 0.01° 2θ.

2) Electron diffraction:

Selected area electron diffraction (SAED) patterns were taken on Sample A using a Philips EM430 transmission electron microscope (with accelerating voltage 300 kV) at 300 ± 2 K and 100 ± 20 K. These [100] zone axis diffraction patterns are fully indexed by an *I*4/*mmm* unit cell (*a* ≈ 3.8 Å, *c* ≈ 16.3 Å) and even with long exposure times, no additional reflections or diffuse scattering was observed. In Bi_2NbO_5F, the NbX_6 (X = O, F) octahedral are rotated about an in-plane axis and about the out-of-plane axis, resulting in an orthorhombic model of *Pbca* symmetry (with a √2*a* × √2*a* × *c* cell) and extra reflections are observed in the [110] electron diffraction patterns. We cannot rule out similar structural distortions in $Bi_2TiO_4F_2$ but further diffraction data would be needed to support this.[1]

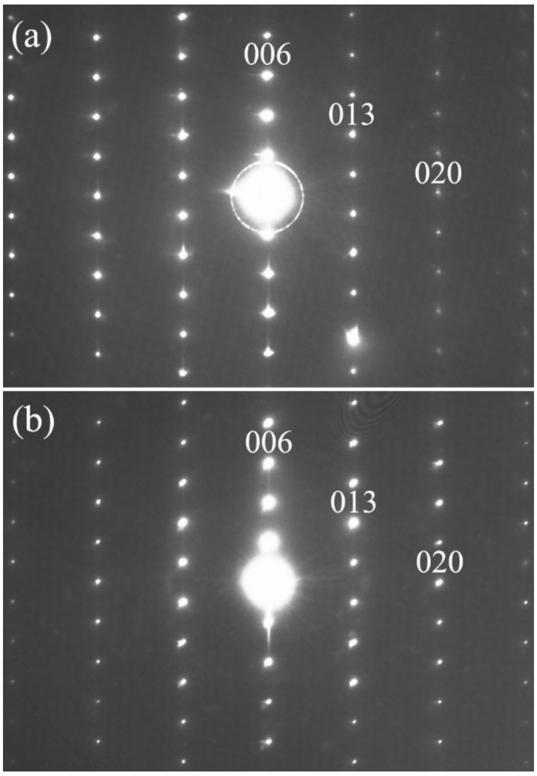

Figure 2: Electron diffraction patterns indexed in [1 0 0] zone axis of *I*4/*mmm*, at a) 100 ± 2 K and b) 300 ± 2 K.

3) Rietveld refinements using 100 K NPD data for Sample A:

Ordered *I*4/*mmm* model with isotropic atomic displacement parameters:

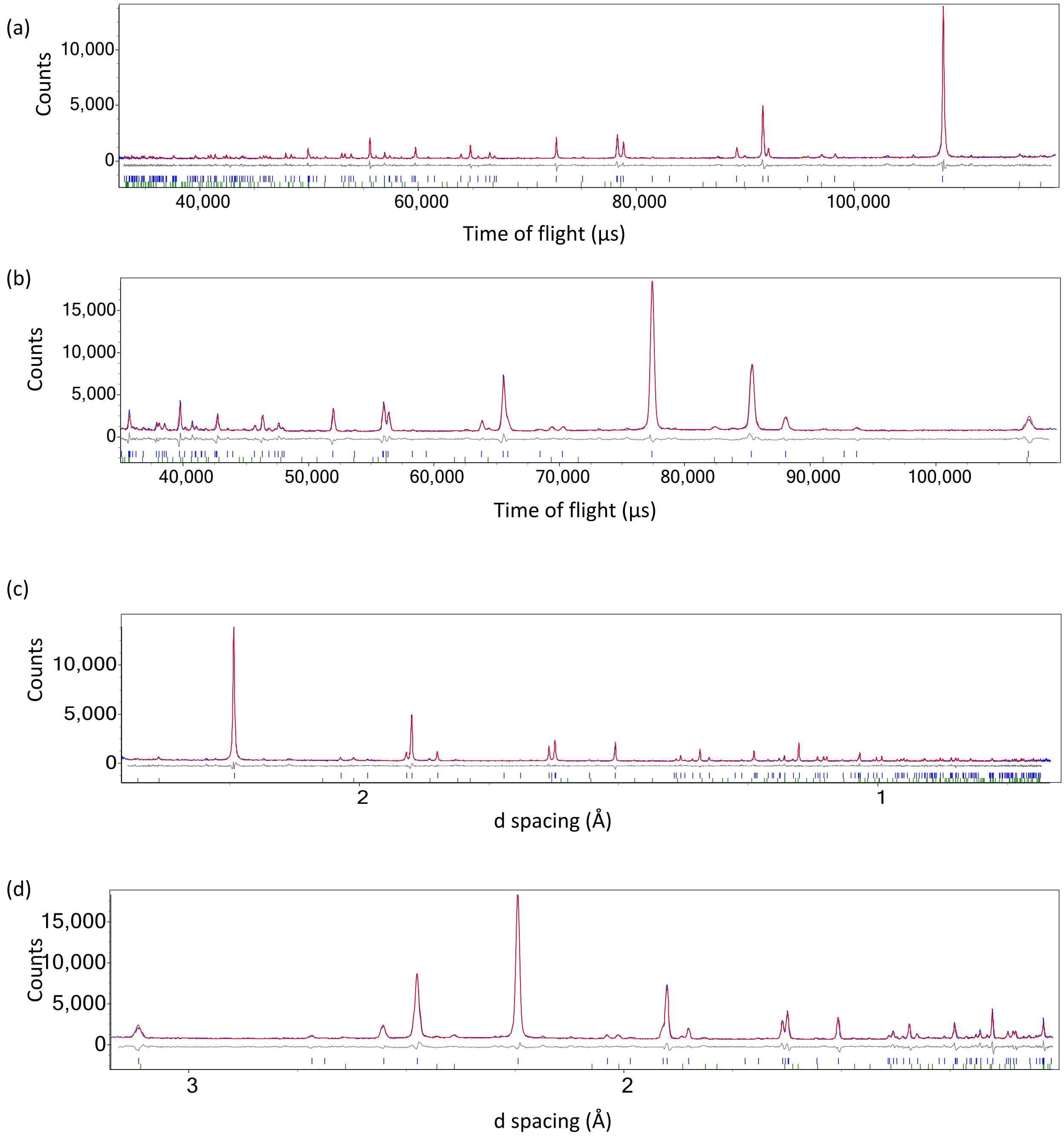

Figure 3 Rietveld refinement profiles using 100 K NPD data and ordered model of *I*4/*mmm* symmetry for $Bi_2TiO_4F_2$ with upper (blue) ticks showing peak positions for main phase (96.1(3)% by weight) and lower (green) ticks showing peak positions for BiOF impurity (3.9(3)% by weight); R_{wp} = 6.95%, R_p = 6.44% (52 parameters). Upper panels (a) and (c) show data from the backscattered band and lower panels (b) and (d) show data from the 90° bank. Observed, calculated and difference profiles are shown in blue, red and grey, respectively.

Table 1 Details from Rietveld refinement profiles using 100 K NPD data and ordered model of *I*4/*mmm* symmetry for $Bi_2TiO_4F_2$; a = 3.80036(7) Å, c = 16.2980(4) Å, volume = 235.38(1) $Å^3$.

**Atom**	**Site**	***x***	***y***	***z***	**U_{iso} × 100 ($Å^2$)**
Bi	4*e*	0	0	0.32785(9)	0.9(1)
Ti	2*a*	0	0	0	3.2(1)
*X*1	4*c*	0	0.5	0	5.6(1)
*X*2	4*e*	0	0	0.1168(1)	3.7(1)
*X*3	4*d*	0	0.5	0.25	0.6(1)

Ordered *I*4/*mmm* model with anisotropic atomic displacement parameters:

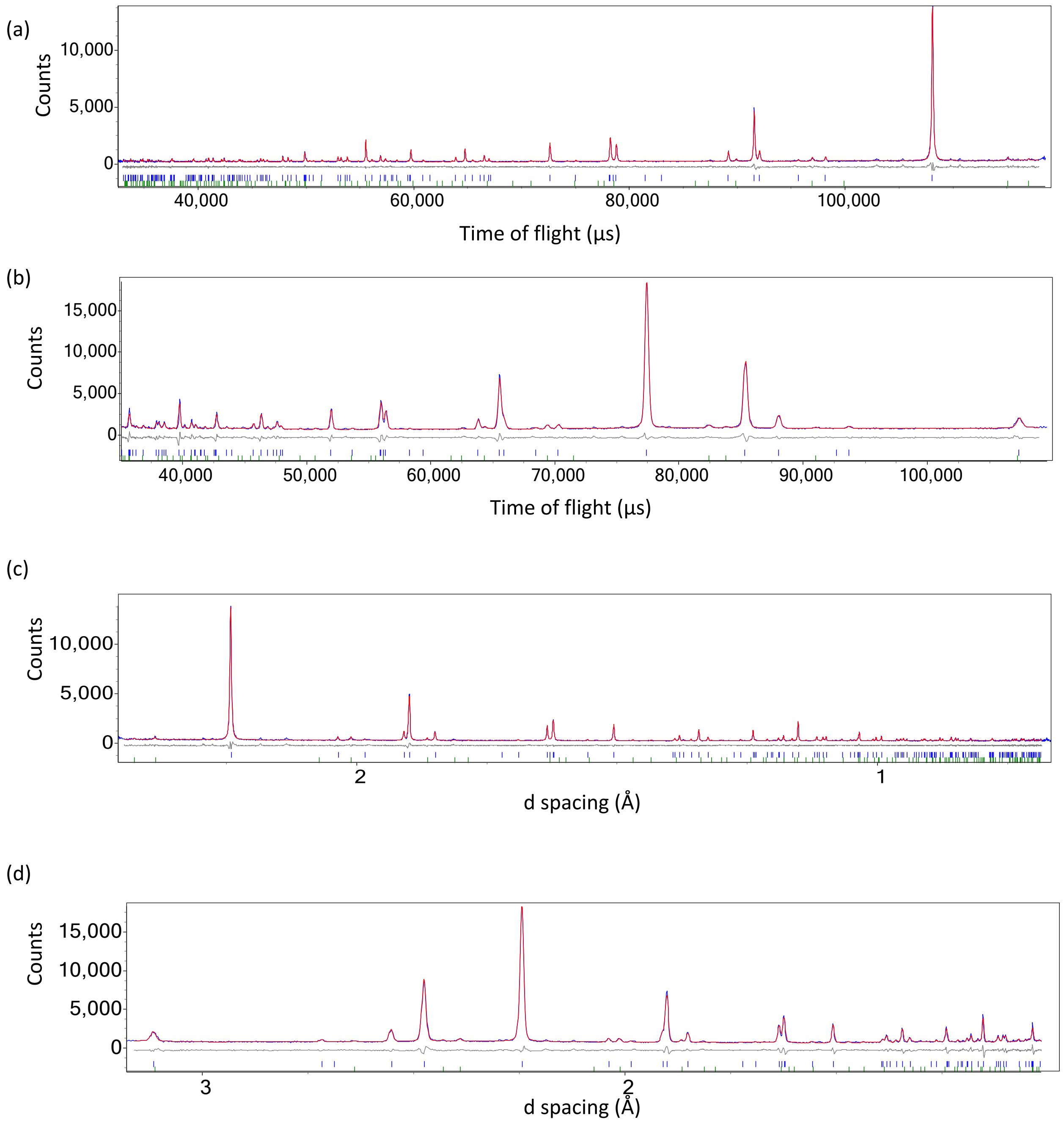

Figure 4 Rietveld refinement profiles using 100 K NPD data and ordered model of *I*4/*mmm* symmetry with anisotropic atomic displacement parameters for *X*1 and *X*2 sites for $Bi_2TiO_4F_2$ with upper (blue) ticks showing peak positions for main phase (96.1(3)% by weight) and lower (green) ticks showing peak positions for BiOF impurity (3.9(3)% by weight); R_{wp} = 6.41%, R_p = 5.86% (55 parameters). Upper panels (a) and (c) show data from the backscattered band and lower panels (b) and (d) show data from the 90° bank. Observed, calculated and difference profiles are shown in blue, red and grey, respectively.

Table 2 Details from Rietveld refinement profiles using 100 K NPD data and ordered model of *I*4/*mmm* symmetry with anisotropic atomic displacement parameters for *X*1 and *X*2 sites for $Bi_2TiO_4F_2$; *a* = 3.80049(7) Å, *c* = 16.2991(4) Å, volume = 235.42(1) Å^3.

**Atom**	**Site**	***x***	***y***	***z***	**U_{iso} × 100 (Å^2)**	**U_{11} × 100 (Å^2)**	**U_{22} × 100 (Å^2)**	**U_{33} × 100 (Å^2)**
Bi	4*e*	0	0	0.32843(9)	0.9(1)			
Ti	2*a*	0	0	0	3.2(1)			
*X*1	4*c*	0	0.5	0		10.3(3)	1.0(1)	7.9(3)
*X*2	4*e*	0	0	0.1168(1)		6.0(1)	6.0(1)	1.4(1)
*X*3	4*d*	0	0.5	0.25	0.6(1)			

100 K disordered *I*4/*mmm* model (with isotropic atomic displacement parameters) as in main text (Figure 4):

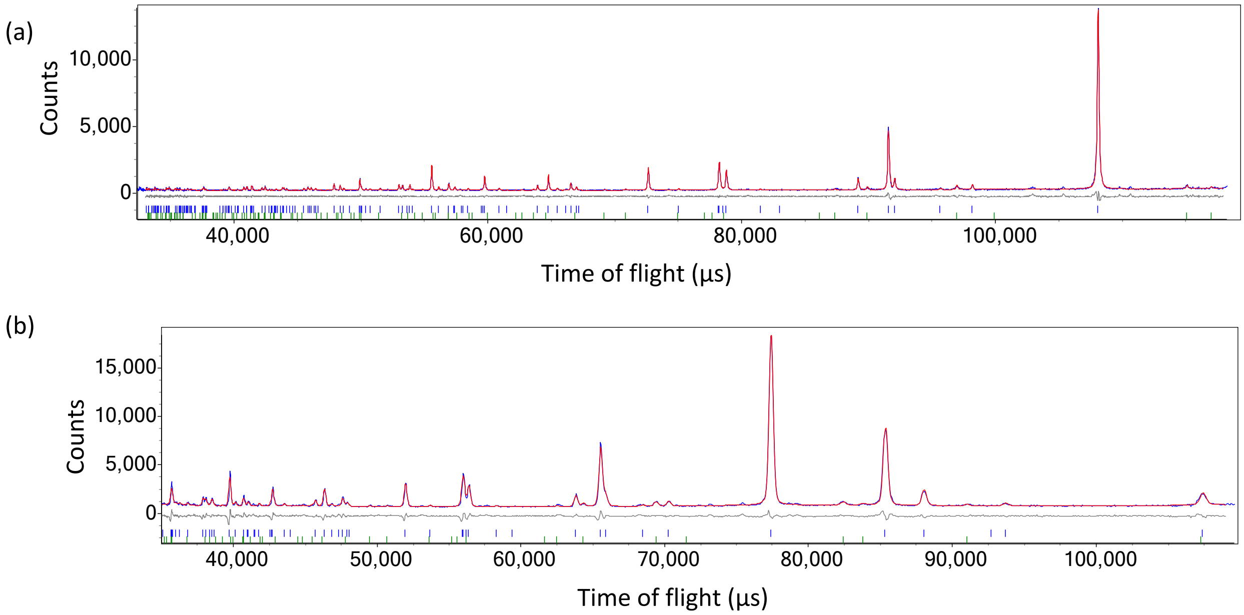

Figure 5 Rietveld refinement profiles using 100 K NPD data and disordered model of *I*4/*mmm* symmetry for $Bi_2TiO_4F_2$ (with displacive disorder of equatorial and apical anion positions to 16*n* and 16*m* sites, respectively) with upper (blue) ticks showing peak positions for main phase (96.1(3)% by weight) and lower (green) ticks showing peak positions for BiOF impurity (3.9(3)% by weight); R_{wp} = 6.39%, R_p = 5.85% (55 parameters). Upper panel (a) shows data from the

backscattered band and lower panel (b) shows data from the 90° bank. Observed, calculated and difference profiles are shown in blue, red and grey, respectively. (This is the equivalent of Figure 4 in the main text but with x axis plotted in time of flight (μs) instead of d spacing.)

4) Common structural distortions for A_2BX_4 and Bi_2BX_6 materials considered for $Bi_2TiO_4F_2$:[2-5]

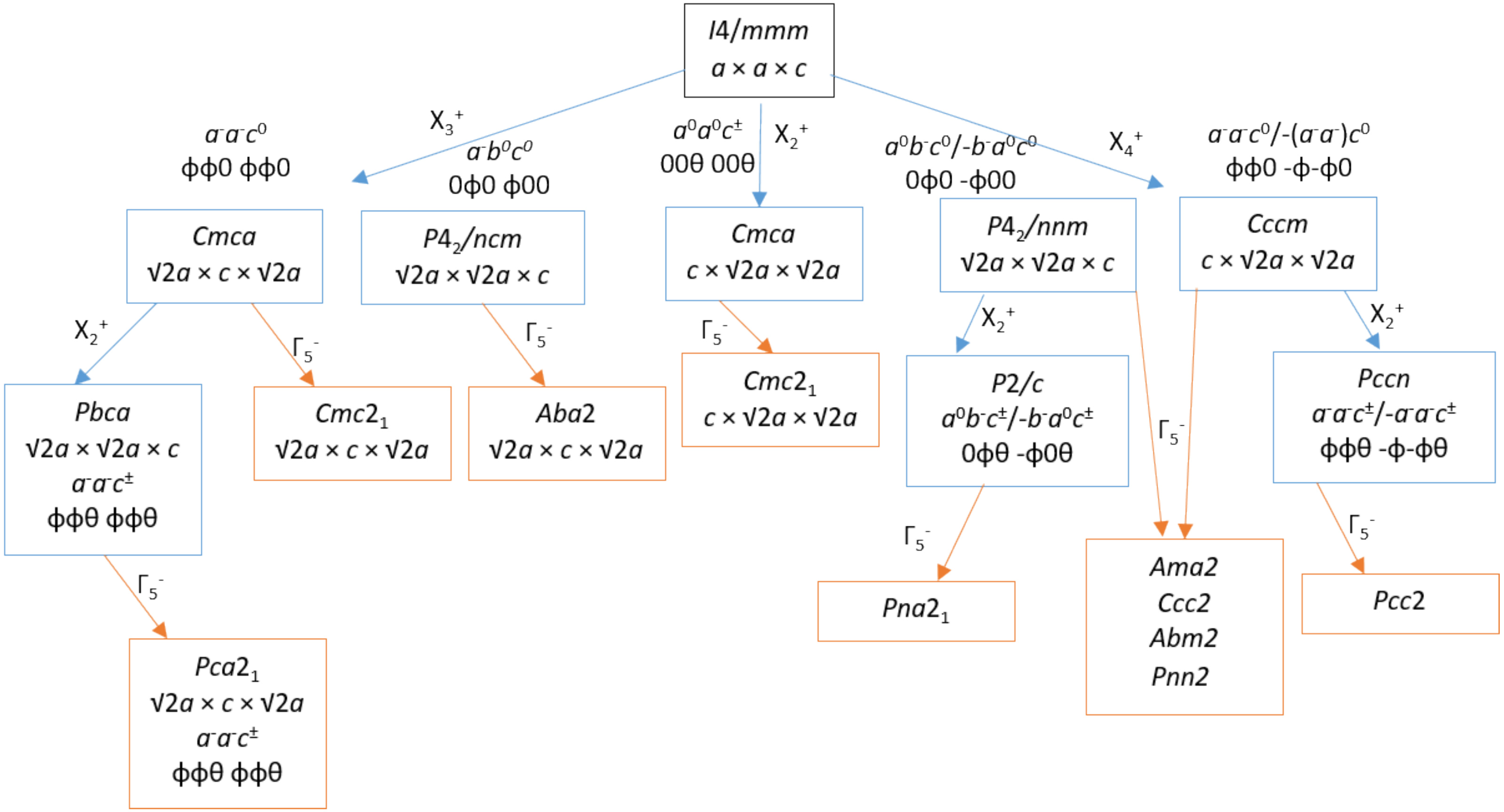

Figure 6 Non-polar distortions (rotations of TiX_6 octahedra) are shown by blue arrows (and non-polar structures in blue boxes), while in-plane polar displacements (Γ_5^-) are shown by orange arrows (and polar structures shown in orange boxes).

X_2^+: rotations of TiX_6 octahedra about the out-of-plane axis ([001] in *I*4/*mmm* parent structure)

X_3^+: out-of-phase rotations of TiX_6 octahedra about an in-plane axis

X_4^+: out-of-phase rotations of TiX_6 octahedra about an in-plane axis

Γ_5^-: in-plane polar displacements

5) Pawley refinements using 100 K NPD data for Sample A to consider larger orthorhombic unit cells:

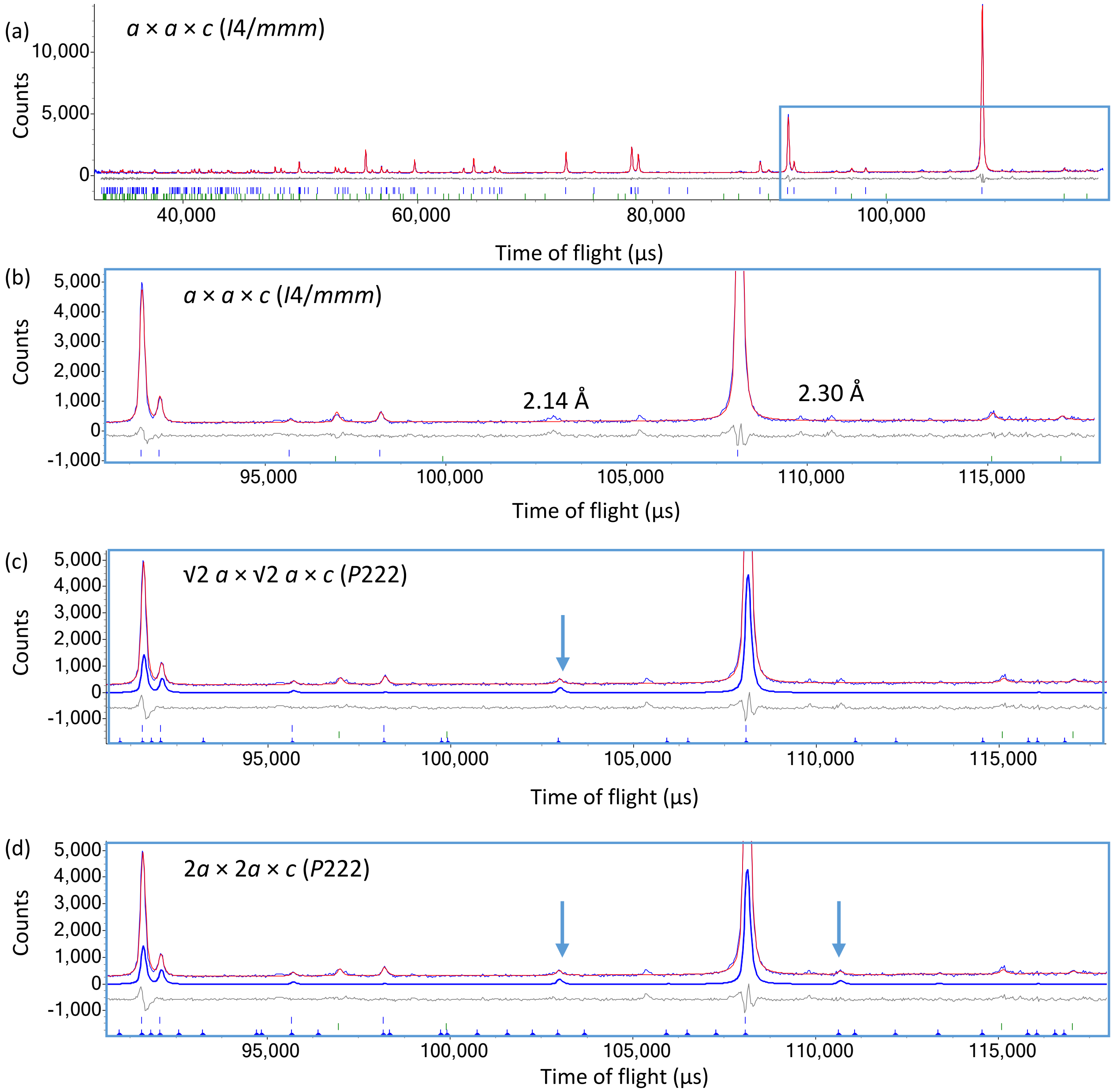

Figure 7 Refinement profiles using 100 K NPD data with a Rietveld model of *I*4/*mmm* symmetry for backscattered bank of data (a) and showing low intensity unindexed peaks in (b), and with a Pawley phase with √2 *a* × √2 *a* × *c* unit cell (c) and 2*a* × 2*a* × *c* unit cell (d). Observed, calculated and difference profiles are shown in blue, red and grey, respectively, and scattering from Pawley phases is highlighted in blue in panels (c) and (d). Ticks show positions of peaks for the $Bi_2TiO_4F_2$ Rietveld phase (top blue), BiOF impurity phase (middle green) and Pawley phase (bottom blue).

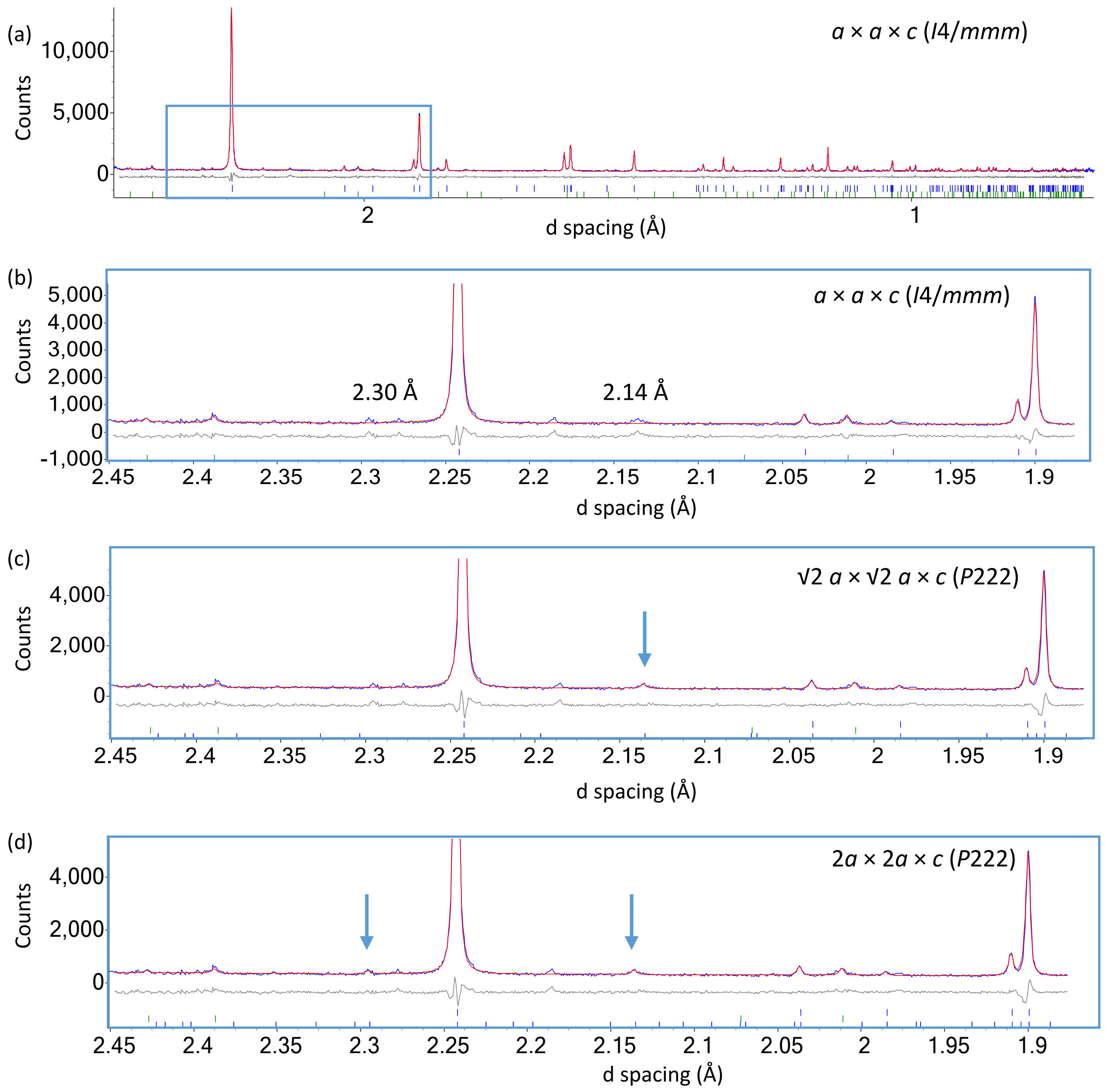

Figure 8 Refinement profiles using 100 K NPD data with a Rietveld model of *I*4/*mmm* symmetry for backscattered bank of data (a) and showing low intensity unindexed peaks in (b), and with a Pawley phase with √2 *a* × √2 *a* × *c* unit cell (c) and 2*a* × 2*a* × *c* unit cell (d). Observed, calculated and difference profiles are shown in blue, red and grey, respectively, and scattering from Pawley phases is highlighted in blue in panels (c) and (d). Ticks show positions of peaks for the $Bi_2TiO_4F_2$ Rietveld phase (top blue), BiOF impurity phase (middle green) and Pawley phase (bottom blue) (Equivalent to Figure 7 but in d spacing rather than time of flight).

6) Mode inclusion analysis

Rietveld refinements were carried out using the high resolution bank of 100 K NPD data to explore possible structural distortions. Distorted structures were described in terms of the parent ordered *I*4/*mmm* model with and the amplitudes of symmetry adapted distortion modes[2, 5] could be refined to describe lower symmetry structures. In our mode inclusion analysis,[6-8] groups of modes were introduced based on their irrep for simulated annealing and the R_{wp} of the best fit was output; the amplitudes of this first group of modes was then fixed at zero and the amplitudes of the next group of modes were refined in simulated annealing. This allowed the improvement in fit from modes of each irrep to be explored.

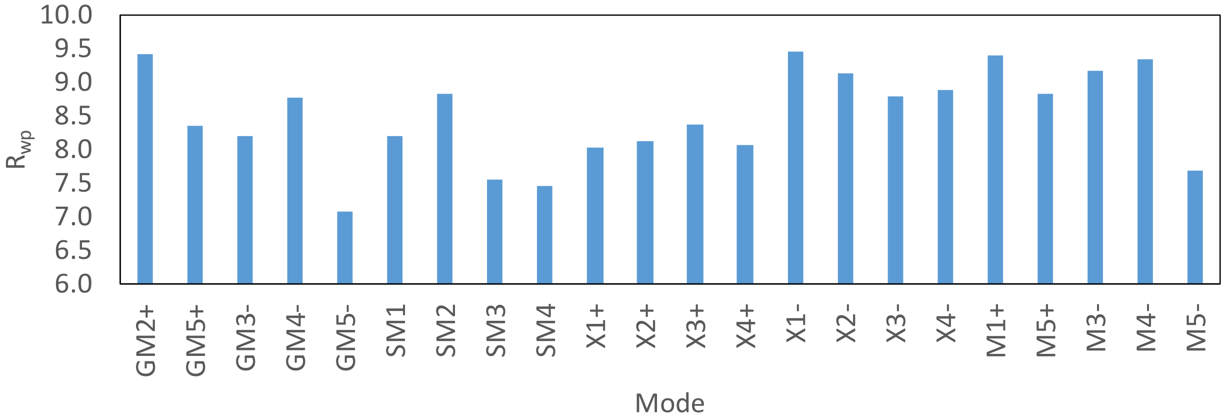

Figure 9 Results of mode inclusion analysis for $Bi_2TiO_4F_2$ using the high resolution bank of 100 K NPD data. The parent ordered structure of *I*4/*mmm* symmetry was used, and the amplitudes of modes (grouped by irrep) were refined using simulated annealing to explore the improvement in R_{wp} for each group of modes.

The results suggested that the most significant improvement is observed when in-plane polar displacements (described by Γ_5^- modes) were allowed.

7) Bond valence sum analysis:[9, 10]

Table 3 Bond valence sum analysis:[9, 10] for $Bi_2TiO_4F_2$ Rietveld using bond lengths from NPD refinement using 300 K disordered *I4/mmm* model.

	**A**<br>***X*1=F, *X*2=O, *X*3=O**	**B**<br>***X*1=O, *X*2=F, *X*3=O**	**C**<br>***X*1=O, *X*2=O, *X*3=F**
Bi	2.92	2.78	2.35
Ti	3.96	4.14	4.35
*X*1	1.23	1.43	1.43
*X*2	1.27	1.04	1.27
*X*3	2.33	2.33	1.76

8) Madelung energy calculations for $Bi_2TiO_4F_2$

Madelung energy calculations on this Bi – Ti – O – F system (Table 4) indicate that the formation of $Bi_2TiO_4F_2$ (for all anion distributions) is enthalpically favourable, but also that the formation of BiOF is also strongly favoured. (No gaseous reagents or products are involved in these reactions and so entropic effects are expected to be relatively minor.) This explains the presence of the BiOF impurity and it is likely that careful temperature control is needed for successful synthesis.

Table 4 Enthalpy changes for solid state reactions determined from Madelung energy calculations for the formation of $Bi_2TiO_4F_2$ (various anion distributions) and BiOF.

**Reaction**	**Enthalpy change ΔH (kJ mol^{-1})**
⅓BiF_3 + ⅓Bi_2O_3 → BiOF	-1740
2BiOF + TiO_2 → $Bi_2TiO_4F_2$ (F_{eq})	+1267
2BiOF + TiO_2 → $Bi_2TiO_4F_2$ (F_{ap})	+7
2BiOF + TiO_2 → $Bi_2TiO_4F_2$ (F_{fl})	+446
⅔BiF_3 + ⅔Bi_2O_3 + TiO_2 → $Bi_2TiO_4F_2$ (F_{eq})	-2212
⅔BiF_3 + ⅔Bi_2O_3 + TiO_2 → $Bi_2TiO_4F_2$ (F_{ap})	-3472
⅔BiF_3 + ⅔Bi_2O_3 + TiO_2 → $Bi_2TiO_4F_2$ (F_{fl})	-3033

9) Symmetry of anion-ordered structures for $Bi_2TiO_4F_2$

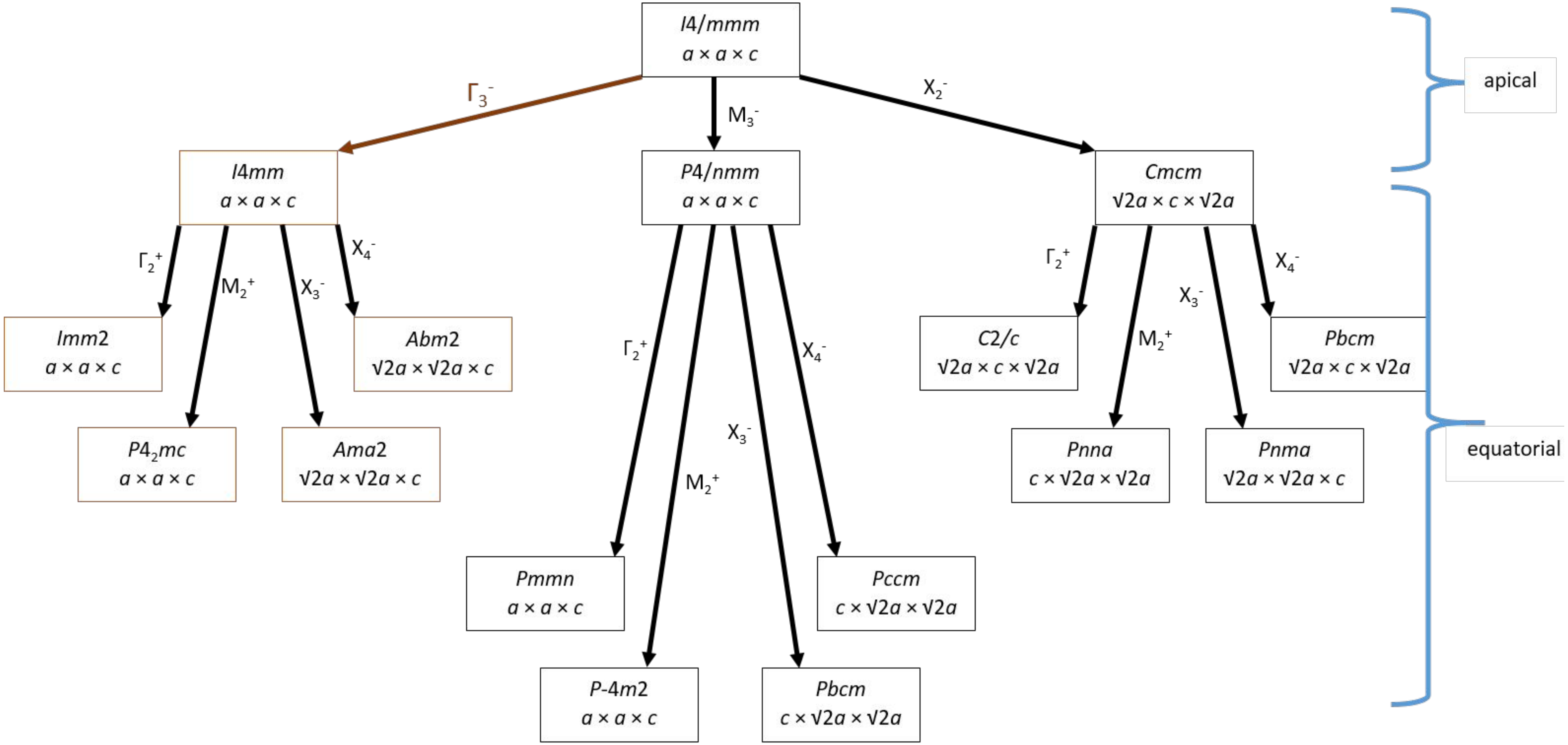

Figure 10 Schematic showing symmetry of anion-ordered structures for $Bi_2TiO_4F_2$ composed of TiO_3F_3 units. Analysis was carried out using ISODISTORT[2, 5] based on the (ordered) parent structure of *I*4/*mmm* symmetry.

Combinations of apical and equatorial occupancy modes to give anion-ordered structures of $Bi_2TiO_4F_2$ containing TiO_3F_3 units:

*Imm*2 ($\Gamma_3^- + \Gamma_2^+$)

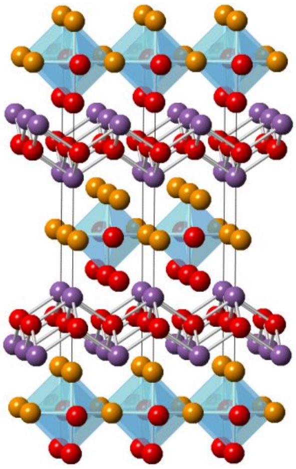

*P*4$_2$*mc* ($\Gamma_3^- + M_2^+$)

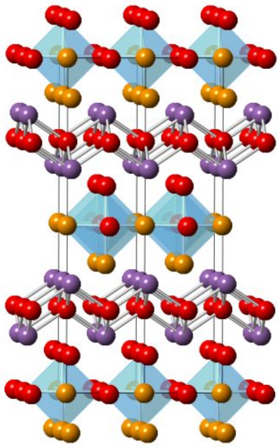

*Ama*2 ($\Gamma_3^- + X_3^-$)

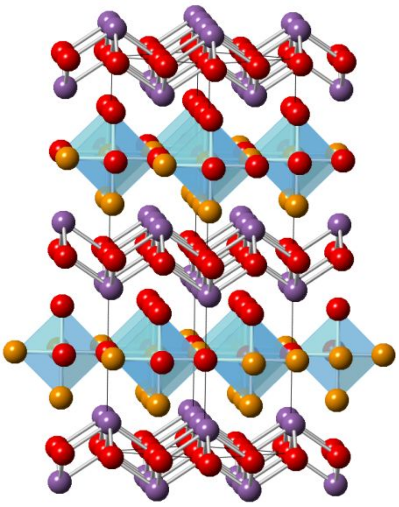

*Abm*2 ($\Gamma_3^- + X_4^-$)

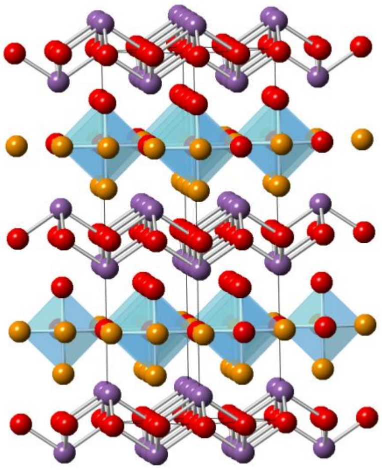

Pmmn ($M_3^- + \Gamma_2^+$)

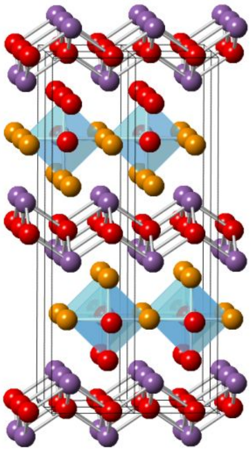

P-4*m*2 ($M_3^- + M_2^+$)

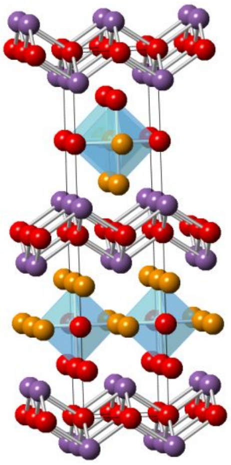

Pbcm ($M_3^- + X_3^-$)

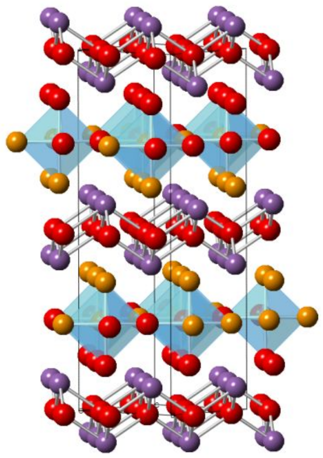

Pccm ($M_3^- + X_4^-$)

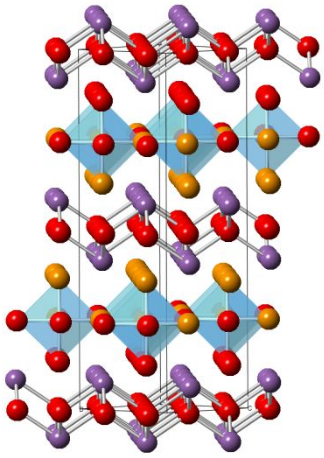

C2/c ($X_2^- + \Gamma_2^+$)

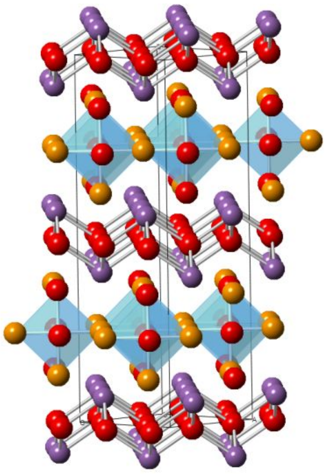

Pnna ($X_2^- + M_2^+$)

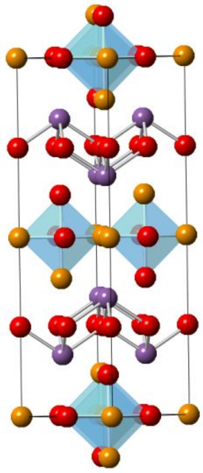

Pnma ($X_2^- + X_3^-$)

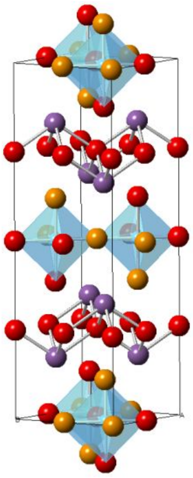

Pbcm ($X_2^- + X_4^-$)

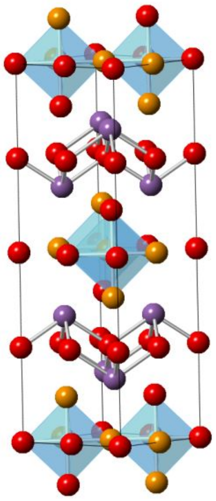

Figure 11 Illustration of anion-ordered structures for $Bi_2TiO_4F_2$ composed of TiO_3F_3 units. Analysis was carried out using ISODISTORT[2, 5] based on the (ordered) parent structure of *I*4/*mmm* symmetry.

10) section from input file for GII refinements

```
str
      a lpa  16.333324`_0.000309
      b lpb  5.385886`_0.000119
      c lpc  5.388397`_0.000112
      al 90.00000
      be 90.00000
      ga 90.00000
      volume  474.014`_0.017
      space_group "Pbcm"
      site Bi1    x xBi1  0.07767`_0.00044 y yBi1  0.49832`_0.00229 z 0.25000     occ Bi   1.00000 beq  bval  0.39938`_0.02607 num_posns  4
      site Bi2    x xBi2  0.57686`_0.00046 y yBi2  0.00141`_0.00198 z 0.25000     occ Bi   1.00000 beq  bval  0.39938`_0.02607 num_posns  4
      site Ti1    x xTi  0.76025`_0.00088  y yTi  0.46240`_0.00236  z 0.25000     occ Ti   1.00000 beq  bval  0.39938`_0.02607 num_posns  4
      site O11    x xO11  0.72965`_0.00049 y 0.25000                z 0.00000     occ O    1.00000 beq  bval  0.39938`_0.02607 num_posns  4
      site F12    x xF12  0.25353`_0.00081 y 0.25000                z 0.00000     occ F    1.00000 beq  bval  0.39938`_0.02607 num_posns  4
      site O21    x xO21  0.86998`_0.00068 y yO21  0.56489`_0.00199 z 0.25000     occ O    1.00000 beq  bval  0.39938`_0.02607 num_posns  4
      site F22    x xF22  0.36243`_0.00061 y yF22 -0.02970`_0.00265 z 0.25000     occ F    1.00000 beq  bval  0.39938`_0.02607 num_posns  4
      site O31    x xO31 -0.00032`_0.00103 y 0.25000                z 0.00000     occ O    1.00000 beq  bval  0.39938`_0.02607 num_posns  4
      site O32    x xO32  0.49948`_0.00099 y 0.25000                z 0.00000     occ O    1.00000 beq  bval  0.39938`_0.02607 num_posns  4
   tof_sample_peakshape( lor_bsl, 0.841391938`_0.0150708154,   dsp_bsl, 56.26705`_0.86144,  !dspsq_bs, 0.00080_1.05354_LIMIT_MIN_0)
      scale scale_bsl  43.5827487`_0.2464
            r_bragg  3.94945611
      Phase_Density_g_on_cm3( 7.95665`_0.00028)

      'Out_CIF_STR(cat_300K_Pbcm_M3-_02.cif)
Get_Distance(Bi1 0 0 0 0 O21 2 1 -1 0, Bi1O211, 2.48608`_0.01595)     '1
Get_Distance(Bi1 0 0 0 0 O21 1 1 1 -1, Bi1O212, 2.84706`_0.00436)     '2
Get_Distance(Bi1 0 0 0 0 O21 2 1 0 0, Bi1O213, 3.16901`_0.01611)      '1
Get_Distance(Bi1 0 0 0 0 O31 3 0 0 0, Bi1O311, 2.29087`_0.01253)      '2
Get_Distance(Bi1 0 0 0 0 O31 5 0 0 0, Bi1O312, 2.28600`_0.01239)      '2

Get_Distance(Bi2 0 0 0 0 F22 1 1 0 0, Bi2F221, 2.87491`_0.00413)      '2
Get_Distance(Bi2 0 0 0 0 F22 2 1 0 0, Bi2F222, 2.71308`_0.01714)      '1
Get_Distance(Bi2 0 0 0 0 F22 2 1 -1 0, Bi2F223, 3.02748`_0.01726)     '1
Get_Distance(Bi2 0 0 0 0 O11 0 0 0 0, Bi2O11, 3.13606`_0.00974)       '2
Get_Distance(Bi2 0 0 0 0 O32 1 1 0 0, Bi2O321, 2.28096`_0.01193)      '2
Get_Distance(Bi2 0 0 0 0 O32 0 0 0 0, Bi2O322, 2.28140`_0.01146)      '2

Get_Distance(Ti1 0 0 0 0 O21 0 0 0 0, TiO21, 1.87537`_0.01800)        '1
Get_Distance(Ti1 0 0 0 0 F22 2 1 0 0, TiF22, 2.00430`_0.01746)        '1
Get_Distance(Ti1 0 0 0 0 F12 3 1 0 0, TiF12, 2.06513`_0.00975)        '2
Get_Distance(Ti1 0 0 0 0 O11 0 0 0 0, TiO11, 1.83661`_0.00938)        '2

Get_Distance(O11 0 0 0 0 Ti1 0 0 0 0, O11Ti, 1.83661`_0.00938)        '2
Get_Distance(F12 0 0 0 0 Ti1 1 1 1 -1, F12Ti, 2.06513`_0.00975)       '2
Get_Distance(O21 0 0 0 0 Ti1 0 0 0 0, O21Ti1, 1.87537`_0.01800)       '1
Get_Distance(O21 0 0 0 0 Bi1 2 1 -1 0, O21Bi11, 3.16901`_0.01611)     '1
Get_Distance(O21 0 0 0 0 Bi1 1 1 1 0, O21Bi12, 2.84706`_0.00436)      '2
Get_Distance(O21 0 0 0 0 Bi1 2 1 0 0, O21Bi13, 2.48608`_0.01595)      '2

Get_Distance(F22 0 0 0 0 Ti1 2 1 -1 0, F22Ti, 2.00430`_0.01746)       '1
Get_Distance(F22 0 0 0 0 Bi2 2 1 -1 0, F22Bi21, 2.71308`_0.01714)     '1
Get_Distance(F22 0 0 0 0 Bi2 1 1 0 0, F22Bi22, 2.87491`_0.00413)      '2
Get_Distance(F22 0 0 0 0 Bi2 2 1 0 0, F22Bi23, 3.02748`_0.01726)      '1

Get_Distance(O31 0 0 0 0 Bi1 1 0 1 -1, O31Bi11, 2.29087`_0.01253)     '2
Get_Distance(O31 0 0 0 0 Bi1 4 0 0 0, O31Bi12, 2.28600`_0.01239)      '2

Get_Distance(O32 0 0 0 0 Bi2 2 1 0 0, O32Bi11, 2.28096`_0.01193)      '2
Get_Distance(O32 0 0 0 0 Bi2 4 0 0 0, O32Bi12, 2.28140`_0.01146)      '2

prm !r0BiO 2.094
prm !r0BiF 1.99
prm !r0TiO 1.815
prm !r0TiF 1.76

prm !VBi 3
prm !VTi 4
prm !VO 2
prm !VF 1
```

```
prm VBi1calc=((Exp((2.094-Bi1O211)/0.37))*1)+((Exp((2.094-Bi1O212)/0.37))*2)+
((Exp((2.094-Bi1O213)/0.37))*1)+((Exp((2.094-Bi1O311)/0.37))*2)
+((Exp((2.094-Bi1O312)/0.37))*2);:  3.02876`_0.02372

prm VBi2calc=((Exp((1.99-Bi2F221)/0.37))*2)+((Exp((1.99-Bi2F222)/0.37))*1)+
((Exp((1.99-Bi2F223)/0.37))*1)+((Exp((2.094-Bi2O11)/0.37))*2)
+((Exp((2.094-Bi2O321)/0.37))*2)+((Exp((2.094-Bi2O322)/0.37))*2);:  2.92408`_0.02231

prm VTicalc=((Exp((1.815-TiO21)/0.37))*1)+ ((Exp((1.76-TiF22)/0.37))*1)
+((Exp((1.76-TiF12)/0.37))*2)+ ((Exp((1.815-TiO11)/0.37))*2);:  4.13159`_0.02996

prm VF12calc=((Exp((1.76-F12Ti)/0.37))*2);:  0.87574`_0.02557

prm VF22calc=((Exp((1.76-F22Ti)/0.37))*1)+((Exp((1.99-F22Bi21)/0.37))*1)+
((Exp((1.99-F22Bi22)/0.37))*2)+((Exp((1.99-F22Bi23)/0.37))*1);:  0.89571`_0.02026

prm VO11calc=((Exp((1.815-O11Ti)/0.37))*2);:  1.88347`_0.04445

prm VO21calc=((Exp((1.815-O21Ti1)/0.37))*1)+((Exp((2.094-O21Bi11)/0.37))*1)
+((Exp((2.094-O21Bi12)/0.37))*2)+((Exp((2.094-O21Bi13)/0.37))*2);:  1.85559`_0.04587

prm VO31calc=((Exp((2.094-O31Bi11)/0.37))*2)+((Exp((2.094-O31Bi12)/0.37))*2);:  2.37472`_0.02442

prm VO32calc=((Exp((2.094-O32Bi11)/0.37))*2)+((Exp((2.094-O32Bi12)/0.37))*2);:  2.41784`_0.02562

penalty = (  VBi - VBi1calc )^2 ;:0.000827154213`
penalty = (  VBi - VBi2calc )^2 ;:0.00576428855`
penalty = (  VTi - VTicalc )^2 ;:0.0173154322`
penalty = (  VO - VO11calc )^2 ;:0.0135787101`
penalty = (  VO - VO21calc )^2 ;:0.0208545277`
penalty = (  VO - VO31calc )^2 ;:0.140416069`
penalty = (  VO - VO32calc )^2 ;:0.174586993`
penalty = (  VF - VF12calc )^2 ;:0.0154393887`
penalty = (  VF - VF22calc )^2 ;:0.0108761055`

prm GII = ((((VBi-VBi1calc)^2)+((VBi-VBi2calc)^2)+((VTi-VTicalc)^2))/3)^0.5;:  0.08927`_0.01004
```

Figure 12 Excerpt from input file for TopasAcademic software for Rietveld refinement using bond valence penalties.

11) Summary of symmetry of anion-ordered structures with tilts[2, 5]

Table 5 Summary of symmetry of anion-ordered structures for $Bi_2TiO_4F_2$ composed of TiO_3F_3 units. Analysis was carried out using ISODISTORT[2, 5] based on the (ordered) parent structure of *I*4/*mmm* symmetry.

Anion-ordered model	with X_2^+ rotations	with X_3^+ rotations	with X_4^+ rotations
Pmmn (*mer*) (M_3^- + Γ_2^+)	*P*2/*c* √2a × *c* × √2a<br>*Cmm*2 2*a* × 2*a* × *c* (Γ_3^-)	*P*2/*c* √2a × *c* × √2a<br>*Cmm*2 2*a* × 2*a* × *c* (Γ_3^-)	*P*2/*c* √2a × *c* × √2a<br>*Cmm*2 2*a* × 2*a* × *c* (Γ_3^-)
P-4*m*2 (*mer*) (M_3^- + M_2^+)	$P222_1$ *c* × √2a × √2a<br>*Cmm*2 2*a* × 2*a* × *c* (Γ_3^-)	$P\text{-}42_1m$ √2a × √2a × *c*<br>$P2_12_12$ √2a × √2a × *c*	$P\text{-}4_2m$ √2a × √2a × *c*<br>*P*222 √2a × *c* × √2a
Pbcm (*fac* (M_3^- + X_3^-)	$Pca2_1$ √2a × *c* × √2a (Γ_5^-)<br>$P2_1/c$ *c* × √2a × √2a	*Pbcm c* × √2a × √2a	*Pma*2 √2a × √2a × *c* (Γ_3^-)<br>*P*2/*c* √2a × *c* × √2a
Pccm (*fac* (M_3^- + X_4^-)	*Pcc*2 *c* × √2a × √2a (Γ_5^-)<br>*P*2/*c c* × √2a × √2a	*Pma*2 √2a × √2a × *c* (Γ_3^-)<br>*P*2/*c* √2a × *c* × √2a	*Pccm* c × √2a × √2a
*C*2/*c* (*mer*) (X_2^- + Γ_2^+)	*C*2 √2a × *c* × √2a (Γ_3^-)<br>*P*2/*c* √2a × *c* × √2a	*Cc* √2a × *c* × √2a (Γ_5^-)<br>$P2_1/c$ √2a × *c* × √2a	*Cc* √2a × *c* × √2a (Γ_5^-)<br>$P2_1/c$ √2a × *c* × √2a
Pnna (*mer*) (X_2^- + M_2^+)	$P222_1$ *c* × √2a × √2a<br>*Pnc*2 √2a × √2a × *c* (Γ_3^-)	$Pna2_1$ *c* × √2a × √2a (Γ_5^-)	*Pnn*2 √2a × *c* × √2a (Γ_5^-)
Pnma (*fac* (X_2^- + X_3^-)	$Pna2_1$ √2a × *c* × √2a (Γ_5^-)<br>$P2_12_12_1$ *c* × √2a × √2a	$P2_1/m$ √2a × √2a × *c*<br>$Pmc2_1$ √2a × *c* × √2a (Γ_5^-)<br>$P2_12_12_1$ √2a × √2a × *c*	$Pna2_1$ √2a × *c* × √2a (Γ_5^-)<br>$Pmn2_1$ √2a × √2a × *c* (Γ_3^-)
Pbcm (*fac* (X_2^- + X_4^-)	*C*2 *c* × √2a × √2a (Γ_5^-)<br>$P2_1/c$ √2a × √2a × *c*<br>$P2_12_12$ *c* × √2a × √2a<br>$Pca2_1$ *c* × √2a × √2a (Γ_5^-)	*Cm c* × √2a × √2a (Γ_3^-) (Γ_5^-)<br>$P2_1/c$ √2a × √2a × *c*<br>$Pca2_1$ *c* × √2a × √2a (Γ_5^-)<br>$Pmc2_1$ √2a × √2a × *c* (Γ_3^-)	*C*2 *c* × √2a × √2a (Γ_5^-)<br>*P*2/*m* 2a × √2a × *c*<br>*Pma*2 √2a × *c* × √2a (Γ_5^-)<br>$P2_12_12$ *c* × √2a × √2a

12) Rietveld refinement with bond valence penalties for anion-ordered $Pca2_1$ model for $Bi_2TiO_4F_2$ using 300 K high resolution NPD data for Sample A

(a)

(b)

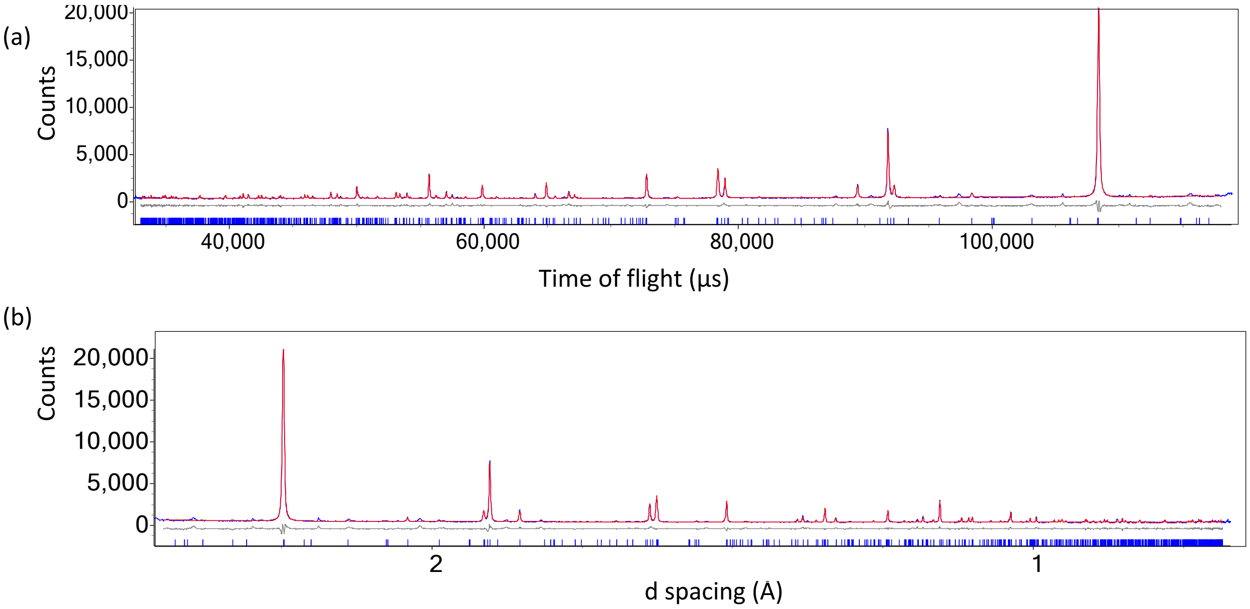

Figure 13 Profiles from Rietveld refinement using bond valence penalties for 300 K NPD data using anion-ordered model of $Pca2_1$ symmetry for $Bi_2TiO_4F_2$; R_{wp} = 6.07%, R_p = 5.73% (55 parameters). Observed, calculated and difference profiles are shown in blue, red and grey, respectively.

Table 6 Details from Rietveld refinement using bond valence penalties for 300 K NPD data using anion-ordered model of *Pca*2$_1$ symmetry for $Bi_2TiO_4F_2$; *a* = 5.3863(1) Å, *b* = 16.3339(2) Å, *c* = 5.3880(1) Å, volume = 474.03(2) Å^3.

**Atom**	**Site**	***x***	***y***	***z***	**U_{iso} × 100 (Å^2)**
Bi(1)	4*a*	-0.010(3)	0.0763(5)	-0.005(7)	0.1(2)
Bi(2)	4*a*	-0.008(3)	0.5762(5)	0.498(7)	0.2(2)
Ti	4*a*	0.02(1)	0.760(1)	0*	3.6(7)
O1	4*a*	0.285(6)	0.733(1)	0.770(8)	1.8(5)
F1	4*a*	0.273(5)	0.256(1)	0.280(1)	0.4(3)
O2	4*a*	-0.009(6)	0.8661(8)	0.010(9)	2.2(4)
F2	4*a*	0.016(5)	0.3652(9)	0.540(7)	1.0(4)
O3	4*a*	0.247(9)	-0.004(1)	0.752(8)	0.3(2)
O4	4*a*	0.246(9)	0.497(1)	0.244(8)	0.2(3)

* Ti *z* coordinate fixed to define origin of the unit cell along the polar axis

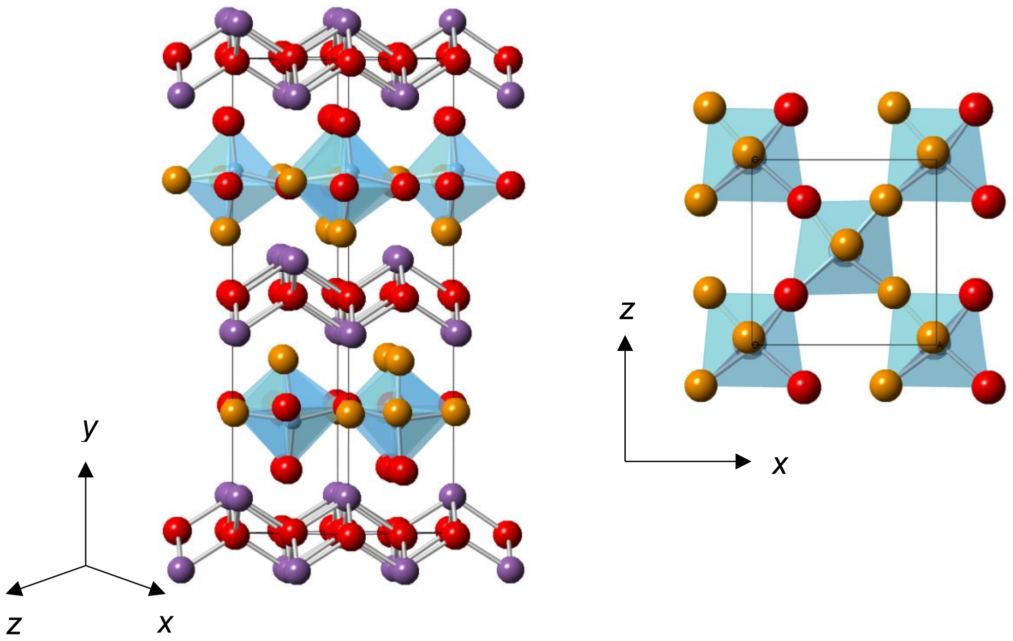

Figure 14 Illustration of the anion-ordered model of *Pca*2$_1$ symmetry for $Bi_2TiO_4F_2$ from Rietveld refinement using bond valence penalties for 300 K NPD data; TiX_6 polyhedra, Bi, O and F ions are shown in blue, purple, red and orange, respectively.

13) Symmetry breaking in anion-ordered n = 1 Aurivillius and Ruddlesden-Popper phases

The $Bi_2TiO_4F_2$ composition discussed above, with hypothetical anion-order with F^- half-occupying apical and equatorial sites, is interesting because this gives rise to corner-linked TiO_3F_3 octahedra, and these *mer*- and *fac*-[TiO_3F_3] units are polar. The occupancy modes discussed above to describe ordering on apical and equatorial sites (Section 9 of this supplementary material) can be applied to other compositions of n = 1 Aurivillius oxyfluorides (and equally to n = 1 Ruddlesden-Popper oxyfluorides which share the same *I*4/*mmm* symmetry for their parent structure, but without the "fluorite" anion site).

Table 7 Summary of symmetries of anion-ordered n = 1 Aurivillius phases assuming an ideal parent structure of *I*4/*mmm* symmetry (and no other structural distortions). The anion sites labelled as "fluorite", "apical" and "equatorial" correspond to Wyckoff sites 4*d*, 4*e* and 4*c*, respectively in the ideal structure of *I*4/*mmm* symmetry. The compositions in this table assume preferential occupancy of the fluorite anion site by O^{2-} anions.

Aurivillius composition Bi_2MX_6	Fluorite site	Apical site	Equatorial site	Corner-linked MX_6 units	Ideal symmetry	
Bi_2MO_6	O_2	O_2	O_2	MO_6	*I*4/*mmm*	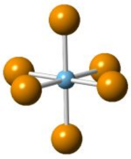
Bi_2MO_5F	O_2	OF	O_2	MO_5F	*I*4*mm* (Γ_3^-)<br>*P*4/*nmm* (M_3^-)<br>*Cmcm* (X_2^-)	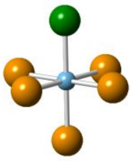
Bi_2MO_5F	O_2	O_2	OF	MO_4F_2	*cis Cmcm* (X_3^-)<br>*cis Cmma* (X_4^-)<br>*trans Immm* (Γ_2^+)<br>*trans P*4$_2$/*mmc* (M_2^+)	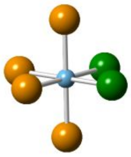<br>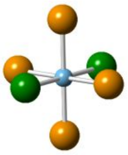
$Bi_2MO_4F_2$	O_2	F_2	O_2	MO_4F_2	*I*4/*mmm*	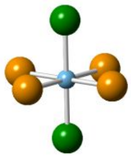
$Bi_2MO_4F_2$	O_2	O_2	F_2	MO_2F_4	*I*4/*mmm*	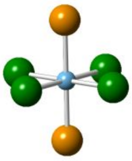

$Bi_2\mathit{M}O_4F_2$	O_2	OF	OF	$\mathit{M}O_3F_3$	See above	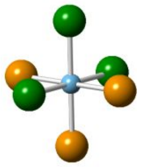 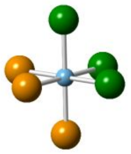
$Bi_2\mathit{M}O_3F_3$	O_2	OF	F_2	$\mathit{M}OF_5$	*I*4*mm* (Γ_3^-)<br>*P*4/*nmm* (M_3^-)<br>*Cmcm* (X_2^-)	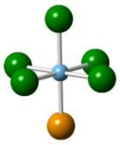
$Bi_2\mathit{M}O_3F_3$	O_2	F_2	OF	$\mathit{M}O_2F_6$	*cis Cmcm* (X_3^-)<br>*cis Cmma* (X_4^-)<br>*trans Immm* (Γ_2^+)<br>*trans P*4_2*/mmc* (M_2^+)	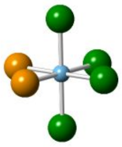
$Bi_2\mathit{M}O_2F_4$,<br>$Bi_2\mathit{M}F_6$	O_2	F_2	F_2	$\mathit{M}F_6$	*I*4/*mmm*	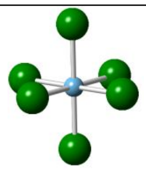

12) Powder diffraction data reporting requirements: crystallographic data (powder)

	100 K	175 K	260 K	300 K
Source	Neutron time of flight	Neutron time of flight	Neutron time of flight	Neutron time of flight
Chemical formula	$Bi_2TiO_4F_2$	$Bi_2TiO_4F_2$	$Bi_2TiO_4F_2$	$Bi_2TiO_4F_2$
Formula weight (g mol^{-1})	567.822	567.822	567.822	567.822
Temperature	100 K	175 K	260 K	300 K
Pressure	ambient	ambient	ambient	ambient
Time of flight data	✓	✓	✓	✓
Crystal system	Tetragonal	Tetragonal	Tetragonal	Tetragonal
Space group number	139	139	139	139
a (Å)	3.80050(6)	3.80313(4)	3.80705(4)	3.80922(4)
b (Å)	3.80050(6)	3.80313(4)	3.80705(4)	3.80922(4)
c (Å)	16.2990(3)	16.3105(2)	16.3247(2)	16.3328(2)
α (°)	90	90	90	90
β (°)	90	90	90	90
γ (°)	90	90	90	90
volume (Å^3)	235.419(9)	235.913(6)	236.604(6)	236.991(6)
Z	2	2	2	2
d spacing range (Å)	2.44 – 0.68	2.44 – 0.68	2.44 – 0.68	2.44 – 0.68
R_{wp} (%)	5.67	4.73	4.66	4.34
R_p (%)	5.56	4.32	4.16	3.97

References

1. McCabe, E. E.; Jones, I. P.; Zhang, D.; Hyatt, N. C.; Greaves, C., Crystal structure and electrical characterisation of Bi_2NbO_5F: an Aurivillius oxide fluoride. *J. Mater. Chem.* **2007,** *17* (12), 1193-1200.
2. Stokes, H. T.; Hatch, D. M.; Campbell, B. J. ISODISTORT, ISOTROPY Software Suite. iso.byu.edu.
3. Hatch, D. M.; Stokes, H. T., CLASSIFICATION OF OCTAHEDRAL TILTING PHASES IN THE PEROVSKITELIKE A_2BX_4 STRUCTURE. *Phys. Rev. B* **1987,** *35* (16), 8509-8516.
4. Hatch, D. M.; Stokes, H. T.; Aleksandrov, K. S.; Misyul, S. V., PHASE-TRANSITIONS IN THE PEROVSKITE-LIKE A_2BX_4 STRUCTURE. *Phys. Rev. B* **1989,** *39* (13), 9282-9288.
5. Campbell, B. J.; Stokes, H. T.; Tanner, D. E.; Hatch, D. M., ISODISPLACE: a web-based tool for exploring structural distortions. *J. Appl. Cryst.* **2006,** *39*, 607-614.
6. Tuxworth, A. J.; McCabe, E. E.; Free, D. G.; Clark, S. J.; Evans, J. S. O., Structural Characterization and Physical Properties of the New Transition Metal Oxyselenide $La_2O_2ZnSe_2$. *Inorg. Chem.* **2013,** *52* (4), 2078-2085.
7. McCabe, E. E.; Stock, C.; Rodriguez, E. E.; Wills, A. S.; Taylor, J. W.; Evans, J. S. O., Weak spin interactions in Mott insulating $La_2O_2Fe_2OSe_2$. *Phys. Rev. B* **2014,** *89* (10).
8. Djani, H.; McCabe, E. E.; Zhang, W.; Halasyamani, P. S.; Feteira, A.; Bieder, J.; Bousquet, E.; Ghosez, P., $Bi_2W_2O_9$: A potentially antiferroelectric Aurivillius phase. *Phys. Rev. B* **2020,** *101* (13).
9. Brown, I. D.; Altermatt, D., BOND-VALENCE PARAMETERS OBTAINED FROM A SYSTEMATIC ANALYSIS OF THE INORGANIC CRYSTAL-STRUCTURE DATABASE. *Acta Cryst. B* **1985,** *41* (AUG), 244-247.
10. Brese, N. E.; O'Keeffe, M., BOND-VALENCE PARAMETERS FOR SOLIDS. *Acta Cryst. B* **1991,** *47*, 192-197.
